# Analysis of rat toxicology studies: statistical agreement between virtual and concurrent controls in detecting treatment effects on liver enzymes

**DOI:** 10.3389/fphar.2026.1704002

**Published:** 2026-04-30

**Authors:** Guillemette Duchateau-Nguyen, Matteo Piraino, Dragomir Ivanov Draganov, Pierre Maliver, Wolfgang Muster, Martina Stirn, Paolo Piraino

**Affiliations:** 1 F. Hoffmann-La Roche Ltd, Computational Sciences Center of Excellence, Roche Innovation Center Basel, Basel, Switzerland; 2 Organon SRL, Bucharest, Romania; 3 F. Hoffmann-La Roche Ltd, Pharmaceutical Sciences, Roche Innovation Center Basel, Basel, Switzerland

**Keywords:** concurrent control group, liver enzyme activity, preclinical toxicology, simulation, virtual control group

## Abstract

**Introduction:**

Virtual control groups (VCGs) are proposed as a means to reduce reliance on live animals in preclinical toxicology studies. Despite the potential benefits of VCGs, concerns remain regarding their comparability to traditional concurrent control groups (CCGs), and the risk of introducing bias into preclinical toxicology research. Our simulation focused on observing the statistical consequences of replacing the CCG (fully or partially) with VCGs derived from historical data, assessing their feasibility and limitations, and determining if VCG use alters study conclusions on selected toxicological endpoints.

**Methods:**

We conducted simulations comparing liver enzyme activity in studies using VCGs and those using CCGs. VCGs were generated by simple matching using a limited set of criteria to a large-scale dataset. One hundred VCGs were generated for each reanalyzed study and the percentage of agreement with CCGs was then assessed across a variety of statistical measures for a selection of endpoints, i.e., liver enzymes and bodyweight.

**Results:**

Full agreement was observed in 46.9% of the performed comparisons between VCGs and CCGs, while full disagreement was noted in 2.6% of comparisons. For the remaining instances of partial agreement, the sampled VCGs predominantly aligned with the CCGs. Discrepancies observed did not consistently affect all measured endpoints within a given analyzed study; they were especially noted when CCG enzyme activity levels approached the boundaries of reference intervals, differing significantly from the majority of the other controls in the database. Partial replacement of CCG with VCG improved the overall agreement. The likelihood of reaching a different conclusion for the overall study outcome was considered to be low. Among the statistical methods used for comparing controls versus treated animals, the method based on the calculation of effect size appeared to be the most appropriate to use, especially with our resampling approach.

**Discussion:**

Our results suggest VCGs could be a potential alternative to CCGs. Key next steps that will need to be addressed include enhancing datasets with richer metadata and standardizing variables across studies to facilitate the selection of matching criteria, refining methods for managing outliers and hypothesis testing, and establishing proxy variables for animal growth.

## 1 Introduction

Virtual control groups (VCGs) are part of innovative study designs that support reduction of animals in preclinical research, aligning with the 3R principles of animal research ethics: replacement, reduction, and refinement (Russell and Burch, 1959; Lauwereyns et al., 2024). VCGs can alleviate recent challenges related to the limited supply of large animals, in particular non-human primates for *in vivo* studies (Ackley et al., 2023). Steger-Hartmann et al. (2020) proposed to use historical data to construct VCGs, which could partially or entirely replace the concurrent control group (CCG) used in trials.

While VCGs are a well-established concept in randomized clinical trials, it has not gained widespread acceptance in preclinical regulatory safety testing. Steger-Hartmann et al. (2020) suggested that this could be due to the absence of large, easily accessible animal control datasets. To address this problem, the European initiative eTRANSAFE innovative medicines initiative (IMI) project developed a prototype VCG that reuses data from control groups in legacy *in vivo* studies assembled in a large repository. This approach was formulated to match virtual controls with animals, aiming to replace CCG animals in future studies (Sanz et al., 2023). To further evaluate and advance the concept of VCGs, the transatlantic think tank for toxicology (t4) sponsored a workshop in 2023, bringing together stakeholders from the pharmaceutical and chemical industries, academia, the Food and Drug Administration, contract research organizations (CROs), and non-governmental organizations. The report of this workshop (Golden et al., 2024) encapsulated the efforts of eTRANSAFE to share, curate and harmonize animal control data in a centralized repository, and the initial methodologies to identify optimal matching criteria between virtual controls and treatment arms in studies. This report also briefly outlined the proof-of-concept study that we had previously developed (Duchateau-Nguyen et al., 2022). This study utilized a preliminary list of matching criteria to consider when constructing VCGs, such as species, strain, sex, route of administration, bodyweight, and the time period during which the VCG studies were conducted. This list is similar to the recommended criteria provided by other studies when building VCGs (Steger-Hartmann et al., 2020; Gurjanov et al., 2023; Wright et al., 2023; Palazzi et al., 2024; Sato et al., 2024). It agrees with the study design factors described by Aulbach et al. (2017) that can trigger physiological changes affecting clinical pathology endpoints. Age and bodyweight of the animals are both important factors driving metabolic parameters and drug metabolism (Jackson et al., 2017; Ghasemi et al., 2021); however, precise birth dates are often unavailable for smaller animal species, with age at study initiation typically reported as a range (e.g., 6–8 weeks). Consequently, bodyweight of the animals was used during matching, which is considered as a surrogate of age (Gurjanov et al., 2024a).

The set of matching criteria that should ultimately be used to generate VCGs remains to be defined. The work undertaken by the VICT3R consortium (Steger-Hartmann et al., 2025a) will address this aspect. In the present work, we tested the use of a limited number of matching criteria, excluding animal supplier, test facility or vehicle, which are commonly considered (Gurjanov et al., 2023; Gurjanov et al., 2024a; Gurjanov et al., 2024b). This work also represents one of the first known attempts to remove outliers during VCG generation. This approach was suggested by Golden et al. (2024), who showed how outliers affected the probability of obtaining identical statistical results when replacing VCGs with CCGs in the reanalysis of toxicology studies.

In the present study, we report the results of a simulation which replaces CCG by VCGs in 40 Wistar Han (WH) rat studies that use one CCG and three treated groups with increasing doses. The primary goal is to determine if VCG use alters the study conclusions based on the statistical assessment of changes in selected toxicological endpoints, specifically liver enzymes and bodyweight. In addition, we examine the feasibility and limitations of using VCGs by looking at reference intervals, outliers, and discuss the suitability of the statistical methods commonly used to detect changes in such studies. VCGs were created using a slightly adjusted algorithm from our proof-of-concept study (Duchateau-Nguyen et al., 2022). Compared with the currently published research, the present work does not try to optimize VCG creation, but generates VCG intentionally from a broader background, relaxing some constraints on study specificities. We also test VCGs with animals from historical controls only (full design, i.e., full replacement of the CCG) and VCGs that retain some of the animals included in the original CCG (hybrid design, i.e., partial replacement of the CCG). The success criteria are measured as the percentage of agreement between analysis performed with CCGs and VCGs.

## 2 Materials and methods

### 2.1 High level description of the methodology steps

A large repository of control animals is created to build VCGs, and a subset of this repository is selected to compute the reference intervals for the liver enzymes of interest. A set of 40 studies with all relevant endpoints is then selected for reanalysis. For each of the 40 studies, (1) sampling and matching algorithms are used to build the VCGs, (2) treated animals are compared to controls (CCG and VCG) and treatment effect sizes are estimated, (3) agreement between analysis performed with CCG and VCG are compared, (4) a power analysis with increasing VCG sample size is performed. These steps are further detailed in the sections below.

### 2.2 Data source

Data from preclinical safety studies conducted at Roche and CRO facilities were gathered from an internal repository, and harmonized by standardizing formats, units, and terminology to ensure consistency and compatibility with the SEND format (Standard for Exchange of Non-Clinical Data, a part of Clinical Data Interchange Standards Consortium). Data were further curated by experts to resolve issues related to non-physiological values. A clean database of historical studies was assembled.

The database included data from a total of 1,163 studies, including 16,110 control animals of the rat species, which were collected between 1971 and 2022. When exploring data by animal strain, the WH strain was the most common (752 studies, 11,004 control animals). The Sprague-Dawley (SpD) strain was also commonly used (127 studies, 2,195 control animals). Other strains collectively accounted for 284 studies and 2,911 control animals. A large diversity was observed in the number of studies and animals available when stratified by other parameters. For some parameters (e.g., route of administration) many studies shared the same setting (oral administration was reported in 493 studies and 7,738 animals of the WH strain, and 78 studies and 1,433 animals of the SpD strain). For other parameters, we observed a prevalent setting among the records available (e.g., for the facility, ∼40% of the studies were carried out in-house and another ∼15% were carried out in a preferred facility; for the animal suppliers, the same vendor was used in ∼50% of the studies). However, for other parameters, the study settings were rather unique and shared by fewer studies (e.g., the vehicle showed 119 distinct descriptors over 189 studies).

All animals used in the studies mentioned above were treated according to the guidelines established by the important regulatory bodies (The International Council for Harmonisation [ICH] and The Organisation for Economic Co-operation and Development [OECD] Guidelines, and Directive 2010/63/EU) (OJEU, 2010). Studies were performed in Association for Assessment and Accreditation of Laboratory Animal Care accredited facilities, and the experimental protocols were reviewed and approved by the relevant ethics committees. The procedures were performed under appropriate anesthesia, and post-operative analgesics were administered as needed to ensure animal welfare throughout the study period.

### 2.3 Selection of studies and endpoints to reanalyze

A homogenous group of studies to reanalyze, where the CCG was replaced by VCGs, was extracted from the study database using the following criteria: studies conducted in WH rats from 2002 to 2022, with oral route of administration, and used a standard study design with a CCG (vehicle) and three test compound groups at low (Lo), medium (Mi), and high (Hi) dose levels. We restricted our reanalysis to four endpoints, namely, three liver enzymes (alanine aminotransferase [ALT], aspartate aminotransferase [AST], alkaline phosphatase [ALP]) and bodyweight changes. We selected these endpoints, especially ALT and bodyweight, because they are critical for study conclusions and are routinely assessed in toxicology studies, ensuring adequate amount of data in our internal repository. In the selected studies, all enzymes were measured in serum samples between Day 7 and Day 35. Under these criteria, 126 studies were identified, and were checked for the following:

- Each treated animal had at least one corresponding matched animal in the controls repository
- The number of matches exceeded at least three times the number of treated animals for at least one sex to perform power simulation calculations. We capped the increase at three times the treated group size because larger values would excessively restrict the availability of studies to explore
- The control group included between three and 15 control animals, and a minimum of 12 days for study duration

As a result of this selection process, 40 studies were identified for reanalysis. For each of these studies, differences between treated (TRT) and controls (CCG or VCG) were assessed in statistical terms for the three liver enzymes. Bodyweight changes from baseline were systematically explored separately in each sex for each reanalyzed study.

### 2.4 Reference intervals

Outliers are data points in a group that deviate markedly from the rest of the members of the group (Johnson and Besselsen, 2002). The presence of outliers in a database, and hence possibly in the sampled VCG, could impact its distribution, mean and standard deviation calculation, thus introducing misleading or incomparable results. Given the diversity in measurement conditions across the studies present in the database, we assumed a study-to-study variability would be present, ranging within certain reference intervals. Values falling outside these intervals due to any unknown reason, such as experimental error, analytical errors, data entry errors and unexpected biological responses due to stress, diet or environment, were considered outliers. Therefore, in order to declare a measurement as an outlier, the intervals for “normal” study-to-study variability were investigated for the species, strain and route of administration under consideration.

In line with Clinical and Laboratory Standards Institute (CLSI) EP28-A3c protocol guidelines (CLSI, 2010), three approaches were used to compute the reference interval for ALT, AST and ALP in each sex: 1) arbitrary bounds at the 2.5th and 97.5th percentiles; 2) mean ± three times the standard deviation (SD; between-study variability) extracted from a mixed-effects model (maximum likelihood estimates) of the enzyme activity level as a function of “sex”, with study as a random effect; 3) the 2.5th and 97.5th percentiles of the fitted distribution obtained from a Box-Cox transformation model (Ammer et al., 2021). The reference intervals obtained from the three approaches were compared, and differences in the upper and lower limits between the three approaches were assessed by clinical pathology experts.

The reference intervals were computed on control animals from a subset of studies conducted on WH rats since 1993, where any vehicle was administered by oral route, with a valid measure of the liver enzymes of interest between 7 and 35 days, excluding recovery periods. This set includes 2,977 control animals from 250 studies from our internal repository.

### 2.5 Matching algorithm

The matching algorithm is the base for VCG generation. This algorithm identified animals in the control arms of the full database, which had the same characteristics as animals in the treatment arms of the studies that were reanalyzed. The scope of matching was to control for key parameters that may affect study outcomes. For each study to be reanalyzed, the following parameters and matching criteria were used:

- Animal specific:
  ○ Biological and genetic characteristics: same species, strain and sex
  ○ Nominal day: the nominal day of enzyme measurement was within ±7 days of the nominal day in the study to be reanalyzed; this aligns with the threshold used in Gurjanov et al. (2024b).
  ○ Bodyweight at baseline: ±5 g for each animal in the treated arms of the study to be reanalyzed. Bodyweight gain (in the age range of rats included in the 40 studies analyzed) is typically between 1.5 to 5 g per day (Vázquez-Borsetti, 2024), a ±5 g difference corresponds to a potential age difference of approximately 1–3 days. This difference is regarded as negligible, considering that the age range of animals within a single study commonly spans about 1 week
- Study specific
  ○ Study design: animals from dose ranging/response studies with three dose levels
  ○ Study year: animals were selected from studies within the last 10 years (relative to the year of the study under reanalysis), to limit potential genetic drift and analytical instrument biases
  ○ Route: same route of administration

Neither the bodyweight on the day of enzyme measurement nor the change in weight were used for matching. Animals used in recovery arms or under recovery at the time of lab assessment were excluded.

Among the available matching methods, such as Propensity Score Matching (recently used to generate VCGs (Li et al., 2024)), Exact Matching, Mahalanobis Distance Matching and Genetic Matching, we utilized the Coarsened Exact Matching (CEM) method (Yu, 2023), which allows for the temporary coarsening of numeric data into meaningful groups, thus facilitating exact matching and maximizing balance between matched groups. For the set of matching criteria used in this work, the CEM method produced more balanced groups than a propensity score-based method would, as discussed by King and Nielsen (2019).

### 2.6 Sampling algorithm

From the pool of matched control animals identified by the matching algorithm, a sampling procedure was used to draw *Z* VCGs, with each VCG containing the same number (*N*) of animals as the CCG of the study to be reanalyzed (or multiples of *N* for the power analysis procedure).

**FIGURE 1:**
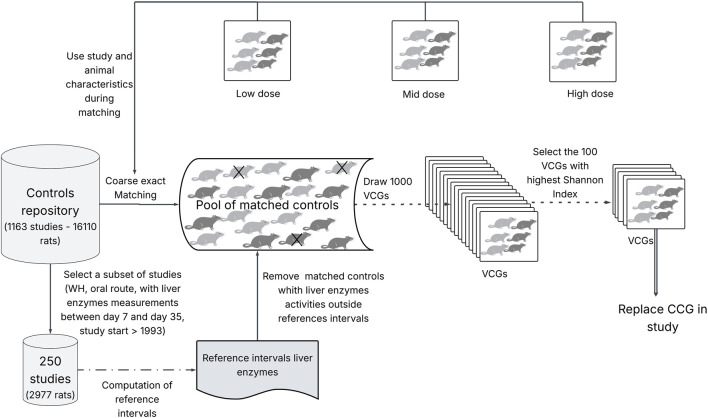
Matching and sampling procedures are depicted in figure above. The number of rat silhouettes illustrates the case of one study with three females (dark grey) and three males (light grey) in each group (control and treated). Matching procedure (depicted by solid line arrows): for the study where VCG will be included, the characteristics of the study (design, year, route of administration) and the animals (species, strain, sex, nominal day of measurements, bodyweight at baseline) are used to identify the corresponding matched animals in the controls repository, using the coarse exact matching algorithm. A subset of the controls’ repository (250 WH studies) is used to compute the reference intervals for each liver enzyme in each sex (see details in Section 2.3). Matched animals whose liver enzyme activities fall outside reference intervals are excluded from the sampling procedure (animals excluded are overlayed with an “X”). Sampling procedure (depicted by dotted line arrows): in a first step 1,000 VCGs are drawn from the pool of matched control animals and a Shannon index is computed for each draw to assess the diversity of the studies from which “matched” controls are drawn. Each VCG contains the same number of animals of each sex as the CCG to be reanalyzed. In a second step, a subset of 100 VCGs with the highest Shannon index values are selected to replace the CCG. If there are more than 100 draws with identical Shannon indexes, VCG are randomly selected.

*Z* was set to 1,000 draws without replacement of size *N*. Selecting 1,000 draws ensures a substantial and representative sample of the possible matched animals. Duplicate draws (containing identical animals), if any, were removed. Only a subset of Z draws were used as VCGs; a sampling procedure was applied, which was initially presented by Duchateau-Nguyen et al. (2022) and later described by Golden et al. (2024). The sampling procedure ensured diversity in the studies' origins, rather than using a random approach. This was completed with the aim to simulate a large repository of studies drawn from multiple companies, thereby reducing the potential for bias stemming from a VCG built solely from animals originating from one study. For each draw, the Shannon diversity index (Shannon, 1948) was calculated for the variable STUDYID (study identifier) to assess the diversity of the studies from which “matched” controls are drawn. The 100 VCGs with the highest Shannon index were then selected for reanalysis. If there are more than 100 draws with identical Shannon index, VCG are randomly selected. The matching and sampling procedures are depicted in Figure 1.

For full CCG replacement in a given study to be reanalyzed, 100 VCGs (full design) are constructed solely from animals drawn from the pool of matched controls, excluding any animals from the original CCG. For partial replacement, 50% of the animals, selected at random, included in each of the 100 VCGs are replaced with animals randomly drawn from the CCG originally included in the study, thus giving 100 h-VCGs (hybrid design).The number of CCG animals is rounded down when the sample size is odd.

### 2.7 Pairwise comparisons - effect size estimations

For each enzyme and each study, data were split based on sex prior to hypothesis testing, according to common practice in toxicology studies. Data normality was assessed for each arm using the Shapiro-Wilk test (the null hypothesis of normally distributed data is rejected if the corresponding p-value is less than 0.05). The assumption of homogeneity of variances was evaluated using Levene’s test when a group did not follow a normal distribution, and Bartlett’s test was used when all groups were normally distributed. Means and SDs of enzyme activity levels were computed for all arms in each study to be reanalyzed, and for all sampled VCG groups (100 samples per study).

A non-parametric multiple comparison rank-based procedure was employed to assess differences in the outcomes across the different treatment groups. This procedure which ranks the original observations does not assume a specific parametric form for the underlying distribution, making it suitable for studies with nonnormal distributions and/or small sample sizes. We used the non-parametric procedure available in the *nparcomp* package (version 3.1; Konietschke et al., 2015; Noguchi et al., 2020). The analysis was designed as a Dunnett-type many-to-one comparison, with the VCG or CCG serving as the control. The hypothesis testing was two-sided, meaning a significant difference in any direction (either an increase or decrease) was tested. Simultaneous adjusted p-values and confidence intervals were calculated using different asymptotic approximation methods to account for the correlations among the test statistics, depending on the results obtained from the tests of normality and equality of variance:

- If all groups passed the normality test, and the equality of variance test indicated homogeneity of variances, the asymptotic method was set to “normal” (multivariate normal distribution)
- If any group failed the normality test, but the equality of variances test indicated homogeneity, the asymptotic method was set to “probit” (multivariate transformation using probit function)
- If the equality of variances test indicated heterogeneity, regardless of normality, the asymptotic method was set to “mult.t” (multivariate Satterthwaite t-Approximation)

Effect size was computed as a relative, probability-based measure rather than a difference in medians. Effect size represents the probability that a randomly chosen observation from one group is greater than a randomly chosen observation from another group (probability of superiority and inferiority). The effect size can range between 0 and 1, where 0.5 indicates no effect; values greater than 0.5 indicate a positive effect, and values lower than 0.5 indicate a negative effect. For example, an effect size of 0.3 means there is a 30% probability that a randomly chosen observation from the treatment group is greater than a randomly chosen observation from the control group (or equivalently, a 70% probability that it is less).

The original analysis of the 40 selected studies did not include a dose-response modelling analysis. As dose-response modelling analysis is not yet widely adopted in toxicology (Kappenberg et al., 2023), it was considered to be outside the scope of the present work and not included in our analysis. Data were analyzed using R statistical software (R version 4.3.0 [2023-04-21] - R Foundation for Statistical Computing, Vienna, Austria).

### 2.8 Comparison of analysis results obtained with CCG and VCGs

To understand the agreement between CCGs and VCGs on results obtained from pairwise comparisons, the percent agreement was computed for each of the 40 studies selected for reanalysis using the two following approaches: (1) an “effect-size based” percent agreement was computed as the proportion of sampled VCGs (across the 100 drawn) with an estimated effect size falling within the confidence interval of the effect size computed for comparisons involving CCG; (2) a “p-value based” percent agreement was calculated as the proportion of p-values from the 100 sampled VCGs that share the same significance (p < 0.05 or p ≥ 0.05) as the CCG.

This assessment was also done for the hybrid design (h-VCGs).

### 2.9 Power analysis

A series of simulations were carried out to determine the effect of increased sample size in VCG on the statistical power of the study and the ability to detect a true effect (if one exists). The reference sample size, given by the original size of the control group in the study, was systematically increased by factors of 1.5, 2, 2.5, and 3. For each sample size multiplier, we generated unique sample combinations (without replacement) from the pool of matched controls using the same sampling procedure described in Section 2.6. All simulations related to power analysis were performed using VCGs where no animals from the original CCG are retained (full design).

The power for detecting differences between the treated and control groups was estimated using a Monte Carlo simulation approach that directly replicated the nonparametric multiple comparison analysis employed in the primary statistical analysis. For each sample size scenario, for 10 VCGs sampled among the 100 VCGs with the highest Shannon index, one thousand datasets were generated and analyzed using the nonparametric multiple comparison procedure described in Section 2.7, with significance assessed at the 0.05 level. The power for each dose group was calculated as the proportion of simulated analyses that yielded statistically significant results.

## 3 Results

### 3.1 Studies to be reanalyzed

The 40 preclinical studies that were selected for reanalysis are presented in Table 1. Studies were conducted between 2002 and 2022; one study was 1 week in duration, 33 studies were 2 weeks in duration and six studies were 4 weeks in duration. Seven studies utilized animals from only one sex.

**TABLE 1:** Studies with assessment of subacute toxicity in animals (1-4 weeks) following oral drug administration.

Study reanalyzed			Original size (CCG)		*N* of matched animals (associated studies)		Diversity in study of origin for each VCG	
ID	Year	Duration	F	M	F	M	F	M
A								
592676	2022	2-W	4	​	27 (9)	​	4/4	​
827977	2022	2-W	4	​	27 (9)	​	4/4	​
157449	2018	2-W	4	4	36 (12)	26 (9)	4/4	4/4
60025	2017	2-W	4	4	40 (16)	31 (8)	4/4	4/4
21740	2011	2-W	4	4	84 (28)	95 (28)	4/4	4/4
21742	2011	2-W	6	​	178 (44)	​	6/6	​
21784	2011	2-W	6	6	111 (30)	104 (29)	6/6	6/6
21830	2011	2-W	​	4	​	82 (29)	​	4/4
21839	2011	2-W	8	8	119 (45)	68 (19)	8/8	8/8
21841	2011	2-W	6	6	221 (53)	187 (50)	6/6	6/6
21843	2011	2-W	6	​	158 (47)	​	6/6	​
18661	2010	2-W	4	4	78 (28)	142 (35)	4/4	4/4
20165	2010	2-W	10	10	237 (50)	207 (45)	10/10	10/10
17271	2009	2-W	10	10	148 (35)	217 (50)	10/10	10/10
22802	2009	2-W	10	10	195 (42)	100 (26)	10/10	9/10
17341	2008	2-W	12	12	66 (16)	191 (42)	9/12	12/12
17372	2008	2-W	4	4	140 (38)	140 (43)	4/4	4/4
17289	2007	2-W	6	​	126 (38)	​	6/6	​
17424	2007	2-W	4	4	107 (39)	46 (14)	4/4	4/4
9477	2006	2-W	6	6	96 (26)	146 (41)	6/6	6/6
9576	2006	2-W	8	8	91 (25)	127 (39)	8/8	8/8
9557	2005	2-W	4	4	56 (21)	186 (42)	4/4	4/4
17192	2005	2-W	6	6	162 (45)	207 (50)	6/6	6/6
17193	2005	2-W	6	6	164 (46)	176 (46)	6/6	6/6
10696	2004	2-W	10	​	69 (22)	​	9/10	​
10704	2004	2-W	6	6	130 (39)	52 (16)	6/6	6/6
13859	2004	2-W	5	5	248 (50)	154 (42)	5/5	5/5
13893	2004	2-W	5	5	195 (48)	199 (43)	5/5	5/5
8647	2003	2-W	4	4	181 (42)	181 (38)	4/4	4/4
8975	2003	2-W	10	10	151 (39)	96 (28)	10/10	9/10
9114	2003	2-W	4	4	198 (45)	83 (30)	4/4	4/4
8893	2002	2-W	6	6	151 (38)	153 (33)	6/6	6/6
8895	2002	2-W	6	6	133 (35)	147 (32)	6/6	6/6
10594	2002	1-W	6	6	99 (31)	123 (29)	6/6	6/6
B								
8737	2002	4-W	14	14	146 (26)	99 (21)	12/14	11/14
10574	2002	4-W	12	12	81 (14)	52 (12)	9/12	8/11
17261	2009	4-W	10	10	77 (12)	71 (11)	8/10	8/10
19002	2009	4-W	4	4	61 (20)	38 (12)	4/4	4/4
21855	2012	4-W	10	9	83 (12)	63 (12)	8/10	7/9
22730	2006	4-W	10	10	116 (21)	77 (15)	9/10	8/10

Table 1 contains all studies reanalyzed, the first part includes 1–2 weeks studies and the second part includes the 4-weeks studies. This table summarizes the study (ID), study year, the size of the original control group for each sex, and the total number of animals available in the database of controls matching the animals in the study. The diversity of animals in each sampled VCG in terms of study of origin is given as minimum and maximum observed across the 100 VCG samples per study. CCG, concurrent control group; F, female; M, male; VCG, virtual control group; W, week.

### 3.2 Reference intervals

**FIGURE 2:**
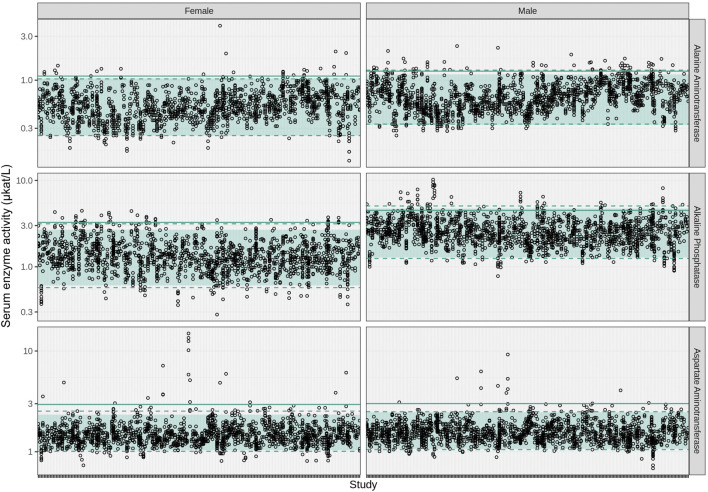
Serum enzyme activity levels in microkatal per liter (μkat/L) for ALT, ALP, and AST plotted for each included study ordered by study year and stratified by sex (study identifiers are not displayed). Each open dot represents the enzyme activity measured in one animal. The shaded regions represent the reference intervals computed using the Box-Cox transformation method, the dashed lines indicate the 2.5th and 97.5th percentiles, and the solid line represents the mean +3 times the between-study variability (SD). ALP, alkaline phosphatase; ALT, alanine aminotransferase; AST, aspartate aminotransferase; SD, standard deviation.

The estimated reference intervals calculated using the three methods described in Section 2.3, and supporting data (enzyme activity levels for ALT, AST and ALP measured in control animals from 250 studies selected from the database) were plotted against each study (ordered by study year) and stratified by sex (Figure 2). The results obtained were comparable; however, the between-study variability approach generated wider reference intervals. This was especially noted for the lower limits, some of which were biologically implausible (e.g., negative values; Supplementary Table 1). In contrast, the two quantile-based approaches produced more consistent and biologically plausible reference intervals. Differences between these two methods were minor, less than 10% for all three enzymes, except for ALT in males and ALP in females. In these two instances, the Box-Cox based method resulted in limits that were 11% (ALT) to 17% (ALP) smaller than those calculated by the other quantile-based method. Overall, differences were considered negligible when examined by toxicology study experts. The Box-Cox transformation method was ultimately chosen due to its robustness and reduced sensitivity to outliers and non-normality.

The reference intervals obtained for ALT ranged from 0.25 to 1.04 μkat/L in females and 0.33 to 1.15 μkat/L in males; ALP ranged from 0.61 to 2.69 μkat/L in females and 1.28 to 4.71 μkat/L in males; and AST ranged from 1.04 to 2.3 μkat/L in females and 1.07 to 2.47 μkat/L in males. In the repository of 250 studies used to compute reference intervals, 710 out of 45,712 measurements (1.6%) were observed outside the reference intervals. These measurements were declared as outliers and excluded from the dataset for building VCGs. Outliers were randomly and equally distributed across sex and laboratory tests (approximately one in every 70 data points in each group). In 88.5% of the animals at a given measurement day, outliers were observed in just one enzyme; in 11.0% and 0.5% of the animals, outliers were observed, for two and three enzymes, respectively, on the same measurement day. Outliers were also found in measurements from control animals in the studies to be reanalyzed; the number of these outliers are found in Supplementary Table 2. These outliers were not removed in the reanalysis and contributed to the variability of CCGs, reflecting what could happen in any study.

### 3.3 Matching

All the animals in the treatment arms of the 40 selected studies had at least one match in the database of controls following the one-to-one matching procedure. The number of matched animals varied widely across studies, from at least three times the size of the CCG in one sex (according to our settings), up to 45 times such size (Table 1).

The matches obtained with the CEM technique shared the same values of the animals in the treatment arm for all discrete parameters used for matching, while study year, nominal day of laboratory measurement and bodyweight at baseline differed within the described ranges in Section 2.4. The average coefficient of variation for nominal day was 17% (i.e., for 2-week studies, the enzymes in VCG animals were measured on average 2.4 days before or after CCG animals).

**FIGURE 3:**
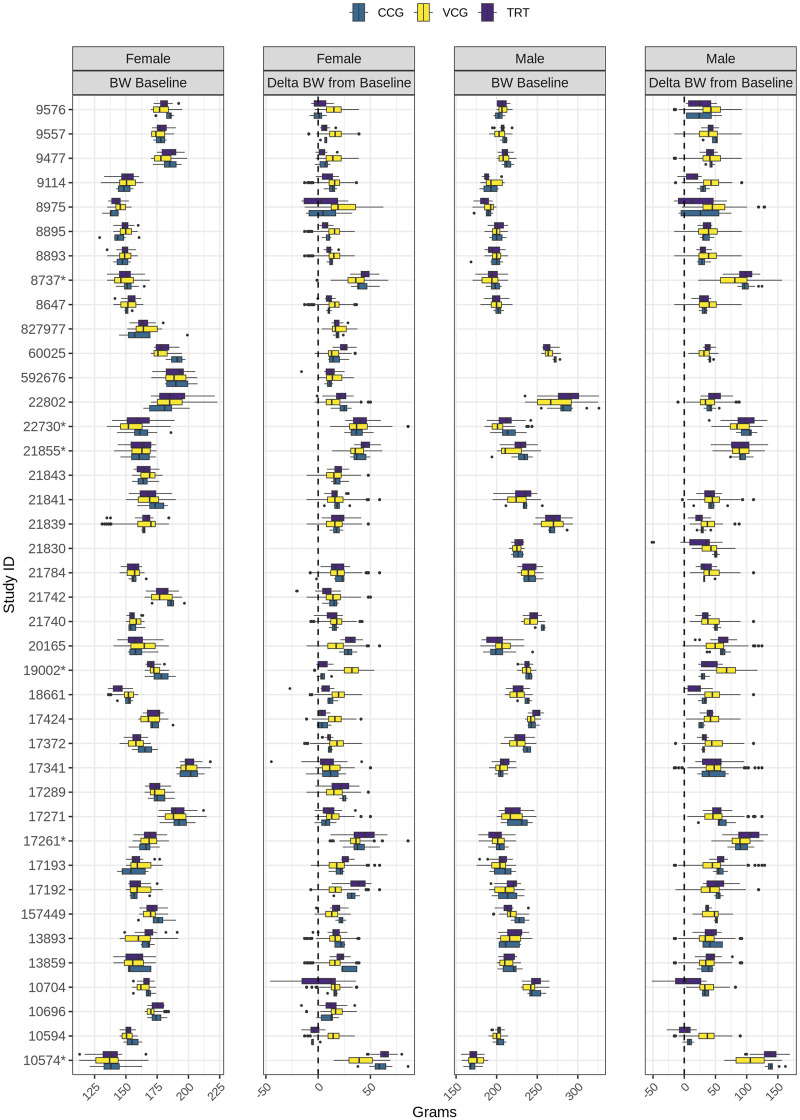
Bodyweight in grams at baseline and difference from baseline to end of treatment for male and female rats across 40 reanalyzed toxicology studies, for CCG (blue), VCG (yellow) and TRT (purple). Median and interquartile ranges are displayed using box plots and outliers are depicted as dots. Studies with an asterisk are 4-week studies, the others are 1-week or 2-week studies. BW, bodyweight; CCG, concurrent control group; TRT, all treatment groups; VCG, virtual control group.

The bodyweight of rats across the 40 studies and all matched animals in each study, at baseline and at the end of treatment, is shown in Figure 3. At baseline, both male and female subjects showed a relatively consistent bodyweight distribution within studies, with minor variations observed between the CCG, VCG, and TRT groups. VCGs covered the whole range of bodyweight seen in TRT groups for all studies as per settings, while CCG showed slightly different distributions in some studies (for example, studies 60025 or 157449).

### 3.4 Descriptive analysis

#### 3.4.1 Normality

Overall, CCG data was normally distributed in the majority of the cases, however, half of the studies reanalyzed did not show normally distributed values for at least one enzyme in one sex group in the CCG. Among the 100 VCGs drawn for each study, enzyme and sex, a median of seven samples (interquartile range [IQR] 6–10)) did not show normally distributed data (Supplementary Table 3). The VCG data shows a very strong tendency to be normal regardless of whether the CCG is normal or not. VCGs showed high normality (>80%) in 92.7% of all cases. This is likely to be related to the outlier removal step.

#### 3.4.2 Average and standard deviations of the endpoints in CCG and VCGs

Across the 40 reanalyzed studies, average bodyweight at baseline in female CCGs and VCGs was 165.8 g (SD = 14.6) and 165.2 g (SD = 13.9), respectively; in male CCGs and VCGs it was 220 g (SD = 25) and 217.8 g (SD = 22.9), respectively (Figure 3). In one study (study ID 10574), the animals were the lightest in bodyweight in both sexes and were approximately 6 weeks old at study initiation. Heavier animals were identified in studies 17341 and 22802 for females and males, respectively. In study 22802, animals were 7–8 weeks old; in study 17341 females were 9–10 weeks old, while males were 7–8 weeks old at study initiation.

The largest differences between bodyweight measured at baseline in the CCG and VCG were in studies 60025 and 21740 for females (mean difference: 11.3 g) and males (mean difference: 13.8 g), respectively.

At the end of treatment, the bodyweight gains of CCG and VCGs were similar in most of the studies in both sexes (Figure 3). In 29 female and 25 male studies, the difference in weight gain between the CCG and VCGs was below 10 g. In the remaining studies, the maximum differences in bodyweight gain were 27.4 g in females and 39.7 g in males. In some cases, bodyweight gains were greater in the VCGs than in the CCG and treated groups, where animals exhibited lower-than-expected growth relative to the other studies (e.g., in study 10594, CCG females lost an average of 4 g, while VCG females gained approximately 20 g). In other cases, bodyweight gains were lower in the VCGs than in the CCG and treated groups, where animals exhibited greater-than-expected growth (e.g., in study 10574, CCG females gained an average of 60 g, whereas VCG females gained an average of 38 g). At the end of treatment, a larger bodyweight gain was observed for all groups and sex, in the 4-week studies compared with the 2-week studies.

**FIGURE 4:**
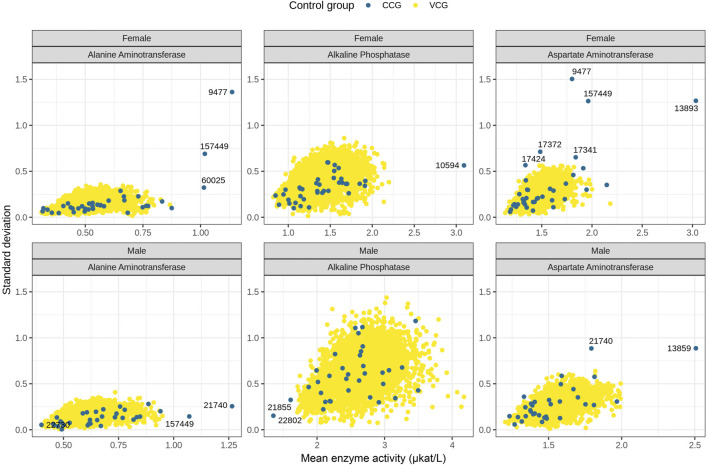
Average enzyme activity levels in microkatal per liter (μkat/L) versus respective standard deviations for CCG (blue dots) and 100 VCGs (yellow dots) across 40 studies. Data for ALT, ALP and AST are presented separately for males and females. Studies with CCG mean enzyme activity levels or their standard deviations out of the range observed for VCGs are labeled with study numbers. AST values in females in study 13,859 (mean = 11.4) and in males in study 13,893 (mean = 4.7) are not shown. ALP, alkaline phosphatase; ALT, alanine aminotransferase; AST, aspartate aminotransferase; CCG, concurrent control group; VCG, virtual control group.

The mean and SD of enzyme activity levels in CCGs and VCGs for all 40 studies are shown in Figure 4. Results for ALT and AST were similar in both sexes, while higher SD values were observed for ALP in males. The enzyme activities for VCGs are comparable to those of CCG, showing similar magnitude and variability, i.e., the blue and yellow data points overlap in Figure 4. CCGs for some studies exhibited extreme enzyme activity levels; such cases were in studies where outliers were detected (Supplementary Table 2). The impact of outliers detected in CCG analysis is further detailed in Section 3.6.1. VCGs did not show extreme values following outlier removal.

### 3.5 Pairwise comparisons

**FIGURE 5:**
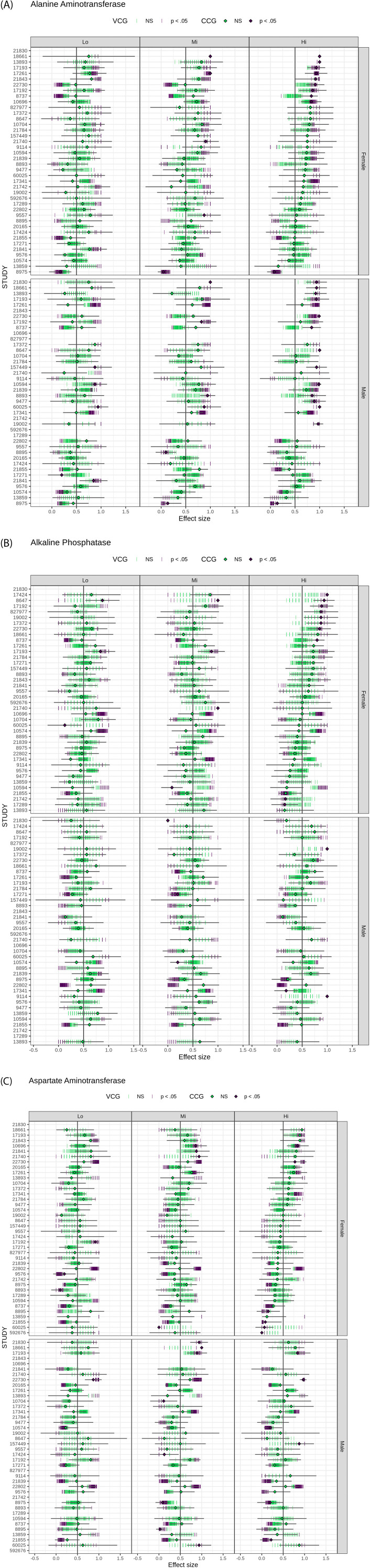
Differences in ALT **(A)**, ALP **(B)** and AST **(C)** activity levels between treated and controls in each study expressed as effect size stratified by sex and treatment groups (Lo, Mi, Hi). Diamonds with horizontal bars represent the effect sizes of the original study using CCGs and their 95% confidence intervals. Vertical bars represent the effect sizes computed for 100 VCGs. The statistical significance (p-values) of the effect is indicated with the same color code for CCG and VCG (green and violet for non-significant and significant changes, respectively). The vertical gray line at 0.5 represents no effect. Studies are sorted by effect size in females who received a high dosage. ALT, alanine aminotransferase; ALP, alkaline phosphatase; AST, aspartate aminotransferase; CCG, concurrent control group; Hi, high; Lo, low; Mi, medium; NS, not significant; VCG, virtual control group.

The effect size obtained for each reanalyzed study of TRT versus control are shown in Figures 5A-C. Results were stratified by enzyme, sex and treatment group. Figure 5 displays the effect size and the 95% confidence interval for the CCG; for the VCGs, only the effect size is displayed (one for each of the 100 VCGs generated).

From the original analysis (using the CCG) of the studies, 10 studies for females and 14 studies for males showed a significant change of ALT activity at the higher dose (Figure 5A) compared with the CCG group. A significant decrease in ALT activity was identified only in study 8975 (across all sexes and dosages, except for male–Lo dosage). Ten studies for females and three studies for males showed a significant change of ALP activity at the higher dose (Figure 5B). The number of studies that reported significant changes in AST activity at high dosages was smaller (six each for both female and male; Figure 5C). Overall, fewer studies showed a change in enzyme activity at low dosages.

Confidence intervals computed on CCGs varied by study. In most studies they were wide enough to cover effect sizes obtained from VCGs, even when a disagreement in statistical significance was observed (e.g., study 21740, female–Mi and Hi dosage; study 15749, female–Mi and Hi dosage; study 17192, male–Mi dosage, study 17372, male–Lo dosage).

### 3.6 Agreement between original results and results obtained with VCGs

**FIGURE 6:**
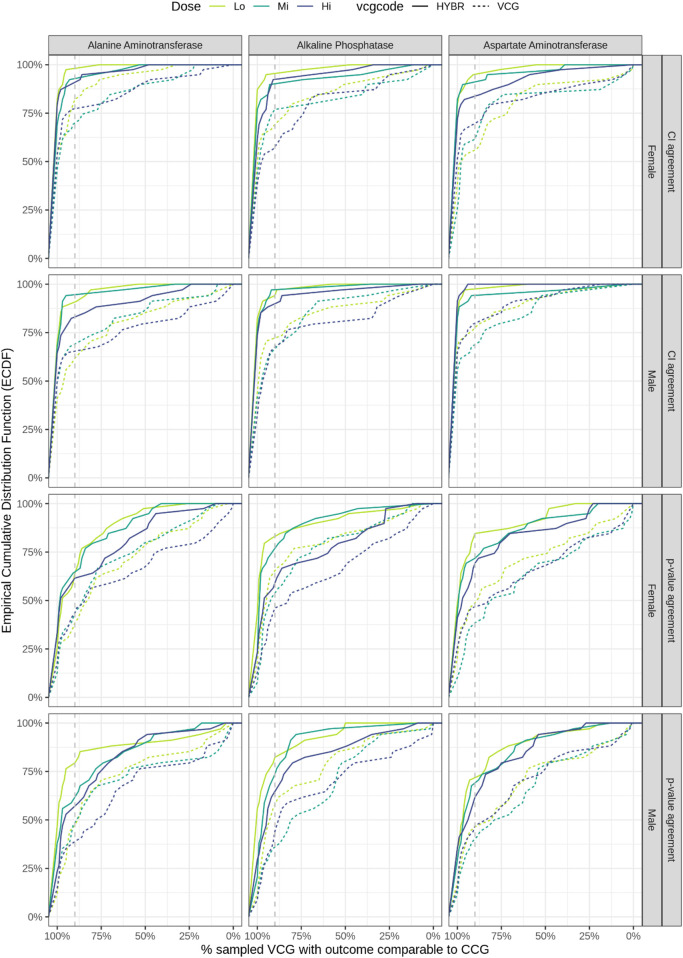
Agreement of pairwise comparisons for the 40 reanalyzed studies. The ECDF displays for each enzyme, sex and dose group (y-axis) the percentage of studies achieving at least X% of agreement between analyses performed with CCGs and those performed with VCGs. The first two rows display the “effect-size based agreement”, while the last two rows display the “p-value based agreement”. The x-axis is reversed (% of agreement decreases from left to right). The dashed and solid lines represent respectively the agreements for VCGs and h-VCGs (HYBR). A grey vertical dashed line at 90% agreement is drawn as reference, i.e., in the high dose group of females for ALT, 75.5% of the 40 studies reanalyzed with VCGs show more than 90% agreement. CCG, concurrent control group; ECDF, empirical cumulative distribution; Hi, high; Lo, low; Mi, medium; VCG, virtual control group; h-VCG, hybrid virtual control group.

If all studies were run with both sexes, there would be a maximum of 720 possible comparisons between treated and controls for the 40 reanalyzed studies (three dosage groups and three enzymes measured in both sex per study). As only one sex was present in seven studies (Table 1), a total of 657 comparisons were performed between controls (CCG or VCG) and treatment groups. The proportion of agreement for these comparisons as an empirical cumulative distribution function (ECDF) of the 40 studies, stratified by enzyme, sex and dose groups, is shown in Figure 6. The threshold of 90% for the studies was used as a reference point for a high percentage of agreement between VCGs and CCGs.

#### 3.6.1 Effect-size based agreement

In 68.5% of all the pairwise comparisons performed there was at least 90% agreement in effect size estimate between analysis done with CCG and VCGs. The highest percentages of agreements were observed for ALT. When stratifying by enzyme, sex and dosage, the percentage of studies with a high percentage of agreement ranged from 53.9% (AST, female–Lo dosage) up to 79.5% (ALT, female–Lo dosage).

In 308 cases (46.9%), there was full agreement between VCGs and CCGs, meaning that the effect size computed for all 100 sampled VCGs fell into the confidence interval computed for the original effect size computed with CCG. Full agreement was found in 34, 31 and 34 studies for ALT, ALP and AST respectively, in at least one dose level and sex. For ALT, ALP and AST there were five studies (10704, 17193, 18661, 21784, 592676), six studies (8975, 9557, 21839, 21843, 592676, 827977) and four studies (10574, 17271, 17424, 21830) with full agreement in all dose groups and in both sexes.

In 2.6% of the pairwise comparisons (17 comparisons from 10 studies, details shown in Supplementary Table 4) there was full disagreement, i.e., none of the effect sizes computed with the 100 sampled VCGs fell into the confidence interval of the effect size computed with CCG. In all 17 cases, enzyme activity levels were either notably higher or lower in CCGs compared with controls from the other studies; some were outside the calculated reference intervals. The distributions for CCG and VCG and the number of outliers detected in CCG are provided in Supplementary Table 2. For study 13859, AST activity levels in female CCGs were above the reference interval computed, leading to full disagreement at all dose levels. Similarly, full disagreement was observed at all dose levels in study 22802 for ALP in male CCGs; in this study, enzyme activity levels were higher in CCGs compared with controls in other studies. For three animals, enzyme activity levels were outside the reference interval computed. In studies 10594 and 13893, for ALP in female rats and AST in male, a full disagreement in at least two dose levels were observed, respectively. In other cases, full disagreement was observed for one enzyme at one dose level and one sex (Figures 5A–C).

Excluding the 17 instances of complete disagreement, and the 308 cases with full agreement, there were 332 cases (50.5%) with partial agreement between VCGs and CCGs. The empirical cumulative distribution in Figure 6 indicates that there was agreement between analysis done with CCG and VCGs in the majority of cases, with 265 instances with at least 50% agreement and 67 instances with less than 50% agreement. The following eight studies exhibited a percentage of agreement equal or lower than 35%: study 8737 for ALT in both sexes, studies 17261, 22802 and 17341 for ALT in males, studies 8647 and 10594 for ALT in females, studies 13859 and 22802 for AST in females, and study 13893 for AST in both sexes.

With h-VCGs, 504 (76.7%) cases with full agreement could be observed and only one comparison (0.15%) was in full disagreement (for AST, study 13859, hi dosage, female).

#### 3.6.2 P-value based agreement

The “p-value based” agreement was lower than the “effect-size based” agreement with VCGs and h-VCGs. At least 90% of agreement was observed in 43.8% of the pairwise comparisons, ranging from 35.3% (ALP, male–Mi dosage) to 61.5% (ALP, female–Lo dosage), with VCG. Agreement was better with h-VCGs than with VCGs, like in the “effect-size based” agreement.

### 3.7 Power analysis

**FIGURE 7:**
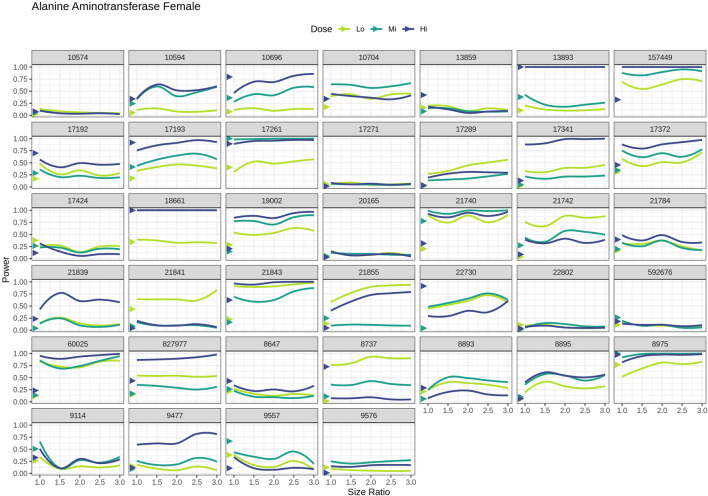
Power for detecting differences in ALT activity levels between the treated and control groups as a function of the ratio of VCG to CCG sample sizes (at a significance level of 0.05), in females per dose group in each reanalyzed study. Triangles indicate the power of the comparison using the CCG as control. A line depicts the power of the comparison using VCGs as control. ALT, alanine aminotransferase; CCG, concurrent control group; Hi, high; Lo, low; Mi, medium; VCG, virtual control groups.

Outcomes derived from power analysis conducted via the augmentation of the sample size for the VCGs revealed significant variance in the reanalyzed studies. However, overarching patterns were consistent across various enzymes. Results for ALT levels in female animals are shown in Figure 7. Further data regarding ALT, AST and ALP levels in males and females are shown in Supplementary Figure 1.

The power calculated for CCG in each study exhibited considerable variability across the respective dose groups, and distinct values were noted across studies. Yet, in the majority of studies, these values remained below 0.25; the highest power was noted for the “Hi” dose group, followed by “Mi”, then “Lo”.

When looking at the power computed for VCGs at varying sample sizes, the expected trend (i.e., power at equal sample size was similar to the values recorded for CCG and increased with incremental sample size increases) was only observed in a subset of studies. Conversely, in other studies, these trends were replaced by a relatively flat trajectory. In some studies, initial values did not align with those recorded for CCGs, which agrees with the expected discrepancies in data distributions between VCG and CCG. This observation suggests that the impact of sample size enlargement on statistical power may be sensitive to the unique conditions and outcomes of specific studies. Analogous findings were documented for AST and ALP, irrespective of sex.

## 4 Discussion

### 4.1 Context and VCG creation procedures

Various sources of variation can influence study endpoints and should ideally be accounted for during matching to enhance the similarity between VCGs and CCGs. For example, Gurjanov et al. (2023), Gurjanov et al. (2024a), Gurjanov et al. (2024b) incorporated species, strain, sex, route of administration, study year, test facility, animal supplier, vehicle, and bodyweight (as a surrogate for age) in their matching criteria for rodent studies. Andaya et al. (2024) further expanded on these by including blood collection methods and frequency, fasting status, and clinical pathology assays. In contrast, our simulation used a restricted set of matching criteria. While certain criteria such as species, strain, sex, and age are known to have an impact on study endpoints (Sato et al., 2024), the effect of other criteria remains unclear. Palazzi et al. (2024) identified 52 matching criteria that could potentially affect endpoint variability, noting that their relative importance is yet to be determined. The ongoing work by the VICT3R consortium (Steger-Hartmann et al., 2025a) is expected to help identify which variables are critical for matching and which can be excluded.

Selecting matching criteria presented difficulties due to the high amount of missing data for certain variables. The initial number of animals of the same species available in the database was reduced by 30% after filtering by strain, and by an additional 30% after filtering by route of administration. Considering that the most common study settings were used, such numbers highlight the importance of using a large and diverse database of historical controls in order to apply the VCG concept based on matched animals, especially if the VCGs will account for multiple study settings and animal characteristics.

It was also difficult to include certain matching criteria due to the lack of harmonization of the terms used. For example, vehicle formulation, a likely important criterion causing toxicity in certain cases (Aulbach et al., 2017), is only captured in the trial summary of 16% of the studies in our repository, and non-standardized terminology further prevented its use. In an internal study using a medium-chain triglyceride (Miglyol 812) as a vehicle, many clinical pathology parameters concentrations were significantly impacted (e.g., increased cholesterol, decreased total protein, and decreased globulins; data not shown), similar results were reported by Sellers et al. (2005). Ongoing collaborative efforts to standardize and harmonize variables (in SEND format) for VCG use (Phuse 2024) are expected to simplify the future utilization of these variables in matching procedures.

Our VCG creation procedure is based on a multiple sampling approach, which maximized the number of studies from which the VCGs originated. This method, initially described in Duchateau-Nguyen et al. (2022) and Golden et al. (2024), allowed us to compensate for the variability of the endpoints measured in the animals included in VCGs. With a limited number of matching criteria, it is likely that created VCGs are still dissimilar to the original CCG. By drawing multiple VCGs and repeating the analysis for each created VCG, our conclusions are much more robust and mitigate the effect of study-specific sources of variation which are known to affect the replicability of *in vivo* studies, as described by Steger-Hartmann et al. (2025b).

### 4.2 Replacing CCGs with VCGs: differences and similarities in terms of statistical outcomes

The identification of the no observed adverse effect level (NOAEL) and the lowest observed adverse effect level (LOAEL) for the drug tested in each of the reanalyzed studies is out of scope for this paper. NOAEL or LOAEL determination requires human assessment based on a variety of endpoints, including non-quantitative parameters such as histopathology. Our study aimed to systematically and objectively explore, through statistical methods, the impact of VCG use on the variability of measured endpoints and the subsequent consequences on the identification of treatment-related effects. None of the 40 reanalyzed studies showed entirely different results in all endpoints tested. Rather, differences were observed occasionally for one or more endpoints in one dose level or sex. In the 2.6% of cases (observed in 10 studies) where full disagreement was observed between analysis performed with VCG and CCG, the enzyme activity levels in CCGs were all either notably higher or lower compared with the control groups in the other studies. Similar insights were drawn by VICT3R partners on SpD rats for hematology endpoints (personal communication, August 2025).

When a larger repository of controls is made available, including studies with richer metadata, it might be possible to identify the reasons for the complete shift of endpoint distributions observed in controls from certain studies. In certain cases, it might be difficult to identify the source of variability. For example, stress is known to influence a number of clinical pathology parameters (Aulbach et al., 2017). Handling procedures, which trigger stress in animals but are not described in the study metadata, may contribute to variability of endpoints measured, depending on the studies sampled to create VCGs. In other cases, it might be possible to reduce the variability observed by considering additional matching criteria (e.g., vehicle), leading to stronger agreement between analyses performed with CCGs and VCGs.

Bodyweight gains were different and more variable in VCGs compared with CCGs in 11 and 15 studies in females and males, respectively. Factors related to the origin of the animals included in the VCGs, such as diet, may affect animal growth. Atypical growth in CCG and treated animals which were observed in some studies (e.g., unusually low in study 10594 and high in study 10574), accounts for the marked differences in body weight gain. The cases that exhibited low agreement for the three liver enzymes tested were not associated with differences in increases in bodyweight between VCGs and CCGs, nor were they associated with high variability of bodyweight across VCGs at the start of the studies. Whilst some endpoints, such as the liver enzyme activity levels, might be differently affected by bodyweight gain (Fujisawa, 2024), other endpoints such as glucose could be affected. Therefore, it could be important to minimize the disparities in bodyweight gain between VCGs and CCGs, by including diet in the matching criteria, and any other factor that could influence animal growth. An alternative would be to identify proxy variables for animal growth, such as food consumption, or use a sufficiently large number of bodyweight measures before dosing starts to predict bodyweight increases, during matching.

### 4.3 Reference intervals and outliers

The reference intervals computed using our database were generally consistent with those reported in the literature (Okamura et al., 2011; Pinches et al., 2019), with minor variations that could be attributed to differences in experimental conditions, population characteristics, and specific instrument methodologies used in different studies. The extreme values present in the repository of historical controls that was used to build VCGs were declared as outliers. As such, they were excluded from the analysis to minimize potential bias. Removal of outliers due to obvious data entry errors is possible (Ludbrook, 2008) and automatic exclusion based on reference intervals could be used in simulation studies and for research purposes, while the handling of outliers in a regulatory context requires confidence on animal health status. As such, additional, study-specific evidence should be considered before excluding any values, as discussed by Friedrichs et al. (2012) and Streiner (2018). The presence of outliers in the CCG was the main driver of full disagreement with the VCG in our simulation; therefore, it is critical to explore the data in the controls repository to be used for VCGs creation and define potential outlying values using reference intervals.

### 4.4 Statistical methods

The objective of statistical analysis may be to test a hypothesis that one or more treated groups is different from the concurrent group; alternatively, the objective may be to estimate the size of an effect in comparison between groups and provide some indication of the precision or confidence that can be ascribed to that estimate (OECD, 2014). Our results indicate that hypothesis testing using a p-value threshold to reject the null hypothesis might be inappropriate to compare outcome data between VCGs and CCGs, especially in settings of low power and lack of biologically relevant changes.

First, the effect size estimates, as described by Kluxen (2020) and Kluxen and Jensen (2021), better reflected the amount of available biological evidence compared with a p-value estimate, and allowed for better characterization of similarities between analysis performed with CCGs and VCGs. Second, hypothesis testing relies on comparing different groups under a specific assumption (null hypothesis), if the experimental conditions change during the data collection process, as is the case when generating VCGs, the assumptions underlying the statistical tests may no longer hold, leading to unreliable or invalid conclusions. In the presence of resampling, the multiple comparisons problem needs to be addressed, and the significance threshold needs adjustment. In this context, the estimation of the size of an effect and its confidence intervals appeared to be a more suitable approach. This is because, when evaluating differences between treated groups and controls, it is of primary importance to consider statistical significance and biological importance, keeping in mind that statistical analysis is part of the interpretation of biological importance, not an alternative (OECD, 2014). Many toxicology studies have been published based on the traditional frequentist approach, particularly on the concept of hypothesis testing and statistical significance. Alternative methods which focus on effect size, confidence intervals, and dose response curves are becoming more accepted in this controversial area (Berselli et al., 2021; Kluxen and Jensen, 2021; Goodman, 1999) and will become more relevant in the presence of new study designs based on the use of historical controls. Bayesian approaches are particularly well-suited in contexts where substantial historical control data are available. Although such methods have been proposed previously (Walley et al., 2016) and practical tools for preclinical scientists are available (Lazic et al., 2018; Bonapersona et al., 2021), their adoption in preclinical research remains limited (Faya et al., 2021; Bradstreet, 2021). Future efforts will focus on integrating Bayesian statistics into the VCG framework for toxicology studies.

The power analysis reported in this study confirmed the need for a more comprehensive and multivariate approach for statistical assessment in preclinical toxicology research. Increasing the sample size of the virtual controls may not necessarily improve agreement between CCGs and VCGs. Our results invite further investigation into factors that can influence statistical power.

### 4.5 Hybrid design

The hybrid design tested here is conceptually similar to the augmented control method approaches previously described by Lin et al. (2019) and Wang et al. (2022), where concurrent and historical controls are combined in the context of clinical trials. Hybrid VCG designs have already been used (Li et al., 2024; Bowman et al., 2026), but no comparison of full design (CCG fully replaced by VCGs) versus hybrid design has been performed so far. The hybrid design (using h-VCG), which incorporates a portion of the CCG animals into the VCG, substantially improved agreement with the original CCG analysis. This effect is a pure statistical consequence of the data overlap. Specifically, full agreement based on effect size increased significantly (from 46.9% to 76.7%), and full disagreement dropped to near-zero (from 2.6% to 0.15%). By sharing 50% of the original data, the h-VCG acts as a statistical anchor for endpoint variability, mitigating the discrepancies that arose when the full VCG was sampled from a historical control distribution that was inherently different from the original CCG’s distribution. This improvement was particularly pronounced in studies where CCG enzyme activity levels approached or crossed the reference range boundaries–cases that suggested underlying, unrecorded study-specific factors. Future work should explore the impact of h-VCGs on the overall interpretation of toxicology studies, especially for CCGs with extreme measurements (outside of reference ranges).

### 4.6 Limitations of our work

A limitation of this study was the small set of endpoints included in our reanalysis. In order to thoroughly examine the results and identify reasons for discrepancies between analyses performed with CCGs and VCGs, we restricted our reanalysis to only three clinical pathology parameters (ALT, AST and ALP) and bodyweight for the selected studies. We acknowledge that the impact of VCGs on study outcome could vary considerably depending on the endpoints analyzed. Further exploration of other categories of endpoints is necessary, such as those related to anatomic pathology assessments or clinical observations and behaviors, which are deemed critical for systemic toxicity testing (OECD, 2014). Preliminary work by Andaya et al. (2024) in one rat study indicates that VCG did not alter the interpretation of the macroscopic and microscopic findings identified in the original study (which utilized CCG). Furthermore, recent work conducted in rat fertility studies demonstrated a generally good concordance between outcomes derived from CCG and VCG (Bowman et al., 2026). Additional investigations across a larger number of studies of various kinds and different species are needed.

Another limitation was the fact that subject matter experts were not involved in defining test article-related effects. Given the substantial number of studies reanalyzed, and the number of VCGs generated for each study, we only used statistical methods to determine the existence and magnitude of the test article-related effects and the difference of effect sizes for both types of controls (CCG or VCG). We are aware that statistics are only one part of the typical analysis performed in toxicology studies, and this analysis alone might not be sufficient to draw a conclusion about an article-related effect and therefore should not replace expert judgments (Ramaiah et al., 2025). Our statistical approach, based on pairwise comparisons, does not account for potential dose-response relationships. Incorporating, when possible, a statistical analysis of dose responses could increase the robustness of the conclusions derived from this analysis (Kluxen and Jensen, 2021) and align more closely with expert assessments of increasing doses.

### 4.7 Future directions

This study highlights several issues that could significantly affect VCG performance in animal studies: 1) outliers and differences in the distribution parameters between VCG and CCG for certain endpoints, 2) variability of animal growth and endpoints and 3) inclusion of matching criteria depends on data availability and quality.

The first two issues may be less critical in large animal [sub]acute studies, where endpoints are measured before treatment starts (baseline values), which could facilitate the identification of outliers in control and treatment groups. To increase agreement between analyses performed with CCG and VCG, matching algorithms based on baseline values could be employed. This approach would exclude VCG animals with baseline endpoint measurements that significantly differ from those in the treated groups. These issues may pose less of a concern in the case of partial replacement of CCG with VCG (hybrid design as defined in Golden et al., 2024), where CCG characteristics could be used during matching.

To address the third issue, future research should focus on developing a database of well-annotated control animals, where study-specific variables are standardized and usable for matching. This is currently under development within the VICT3R consortium (Steger-Hartmann et al., 2025a). Investigating the applicability of VCGs across a large set of endpoints and species will also be critical to generalize the use of VCGs. Finally, collaboration with regulatory bodies to align methodologies and acceptance criteria will be essential for broader adoption of VCGs. As the database grows and methodologies improve, VCGs could become standard for preclinical study designs, reducing reliance on animals and expediting the drug development process.

## Data Availability

The original contributions presented in the study are included in the article/Supplementary Material, further inquiries can be directed to the corresponding author.

## References

[B1] Ackley D. Birkebak J. Blumel J. Bourcier T. de Zafra C. Goodwin A. (2023). FDA and industry collaboration: identifying opportunities to further reduce reliance on nonhuman primates for nonclinical safety evaluations. Regul. Toxicol. Pharmacol. 138, 105327. 10.1016/j.yrtph.2022.105327 36586472

[B2] Ammer T. Schuetzenmeister A. Prokosch H. U. Rauh M. Rank C. M. Zierk J. (2021). refineR: a novel algorithm for reference interval estimation from real-world data. Sci. Rep. 11, 16023. 10.1038/s41598-021-95301-2 34362961 PMC8346497

[B3] Andaya R. Sullivan R. Pourmohamad T. Hayes M. Maliver P. Laing S. (2024). A proof-of-concept rat toxicity study highlights the potential utility and challenges of virtual control groups. ALTEX 41, 647–659. 10.14573/altex.2404201 39132891

[B4] Aulbach A. Provencher A. Tripathi N. (2017). Influence of study design variables on clinical pathology data. Toxicol. Pathol. 45, 288–295. 10.1177/0192623316677066 28178900

[B5] Berselli N. Filippini T. Adani G. Vinceti M. (2021). “Dismissing the use of P-values and statistical significance testing in scientific research: new methodological perspectives in toxicology and risk assessment,” in Toxicological risk assessment and multi-system health impacts from exposure. Editor Tsatsakis A. M. (Elsevier), 309–321. 10.1016/B978-0-323-85215-9.00002-7

[B6] Bonapersona V. Hoijtink H. Relacs C. Sarabdjitsingh R. A. Joëls M. (2021). Increasing the statistical power of animal experiments with historical control data. Nat. Neurosci. 24, 470–477. 10.1038/s41593-020-00792-3 33603229

[B7] Bowman C. J. Li D. Tran T. D. B. Catlin N. R. Stethem C. M. Nowland W. S. (2026). Exploration of virtual control groups and Bayesian approaches for rat fertility studies. Regul. Toxicol. Pharmacol. 165, 106004. 10.1016/j.yrtph.2025.10600 41314368

[B8] Bradstreet T. E. (2021). Breaking the Bayesian ice with preclinical discovery biologists by predicting inadequate animal enrolment. Stat. Biopharm. Res. 13, 344–354. 10.1080/19466315.2020.1799856

[B9] Clinical and Laboratory Standards Institute (CLSI) (2010). Defining, establishing, and verifying reference intervals in the clinical laboratory; approved guideline. 3rd ed. Wayne, PA: CLSI. Available online at: https://clsi.org/shop/standards/ep28/ (Accessed August 14, 2025).

[B10] Duchateau-Nguyen G. Draganov D. Maliver P. Muster W. Piraino P. Piraino M. (2022). “Virtual control groups in animal toxicology studies,” in Non-Clinical statistics conference, louvain-la-neuve. Available online at: https://ncs-conference.org/wp-content/uploads/2022/12/Duchateau-Nguyen-D1-20m.pdf (Accessed August 14, 2025).

[B11] Faya P. Sondag P. Novick S. Banton D. Seaman Jr J. W. Stamey J. D. (2021). The current state of Bayesian methods in nonclinical pharmaceutical statistics: survey results and recommendations from the DIA/ASA-BIOP Nonclinical Bayesian Working Group. Pharm. Stat. 20, 245–255. 10.1002/pst.2072 33025743

[B12] Friedrichs K. R. Harr K. E. Freeman K. P. Szladovits B. Walton R. M. Barnhart K. F. (2012). ASVCP reference interval guidelines: determination of *de novo* reference intervals in veterinary species and other related topics. Vet. Clin. Pathol. 41, 441–453. 10.1111/vcp.12006 23240820

[B13] Fujisawa N. (2024). Understanding the effects of food restriction on toxicological parameters: a comparative analysis in rats, dogs, and monkeys. Fundam. Toxicol. Sci. 11, 123–130. 10.2131/fts.11.123

[B14] Ghasemi A. Jeddi S. Kashfi K. (2021). The laboratory rat: age and bodyweight matter. EXCLI J. 20, 1431–1445. 10.17179/excli2021-4072 34737685 PMC8564917

[B15] Golden E. Allen D. Amberg A. Anger L. T. Baker E. Baran S. W. (2024). Toward implementing virtual control groups in nonclinical safety studies. ALTEX 41, 282–301. 10.14573/altex.2310041 38043132

[B16] Goodman S. N. (1999). Toward evidence-based medical statistics. 1:The P value fallacy. Ann. Intern Med. 130, 995–1004. 10.7326/0003-4819-130-12-199906150-00008 10383371

[B17] Gurjanov A. Kreuchwig A. Steger-Hartmann T. Vaas L. A. I. (2023). Hurdles and signposts on the road to virtual control groups – a case study illustrating the influence of anesthesia protocols on electrolyte levels in rats. Front. Pharmacol. 14, 1142534. 10.3389/fphar.2023.1142534 37153793 PMC10159271

[B18] Gurjanov A. Vaas L. A. I. Steger-Hartmann T. (2024a). The road to virtual control groups and the importance of proper body-weight selection. ALTEX 41, 660–665. 10.14573/altex.2403141 38809255

[B19] Gurjanov A. Vieira-Vieira C. Vienenkoetter J. Vaas L. A. I. Steger-Hartmann T. (2024b). Replacing concurrent controls with virtual control groups in rat toxicity studies. Regul. Toxicol. Pharmacol. 148, 105592. 10.1016/j.yrtph.2024.105592 38401762

[B20] Jackson S. J. Andrews N. Ball D. Bellantuono I. Gray J. Hachoumi L. (2017). Does age matter? The impact of rodent age on study outcomes. Lab. Anim. 51, 160–169. 10.1177/0023677216653984 27307423 PMC5367550

[B21] Johnson P. D. Besselsen D. G. (2002). Practical aspects of experimental design in animal research. ILAR J. 43, 202–206. 10.1093/ilar.43.4.202 12391395

[B22] Kappenberg F. Duda J. C. Schürmeyer L. Gül O. Brecklinghaus T. Hengstler J. G. (2023). Guidance for statistical design and analysis of toxicological dose–response experiments, based on a comprehensive literature review. Arch. Toxicol. 97, 2741–2761. 10.1007/s00204-023-03561-w 37572131 PMC10474994

[B23] King G. Nielsen R. (2019). Why propensity scores should not be used for matching. Polit. Anal. 27, 435–454. 10.1017/pan.2019.11

[B24] Kluxen F. M. (2020). “New statistics” in regulatory toxicology? Regul. Toxicol. Pharmacol. 117, 104763. 10.1016/j.yrtph.2020.104763 32781239

[B25] Kluxen F. M. Jensen S. M. (2021). Expanding the toxicologist’s statistical toolbox: using effect size estimation and dose-response modelling for holistic assessments instead of generic testing. Regul. Toxicol. Pharmacol. 121, 104871. 10.1016/j.yrtph.2021.104871 33485925

[B26] Konietschke F. Placzek M. Schaarschmidt F. Hothorn L. A. (2015). Nparcomp: an R software package for nonparametric multiple comparisons and simultaneous confidence intervals. J. Stat. Softw. 64, 1–17. 10.18637/jss.v064.i09

[B27] Lauwereyns J. Bajramovic J. Bert B. Camenzind S. De Kock J. Elezović A. (2024). Toward a common interpretation of the 3Rs principles in animal research. Lab. Anim. 53, 347–350. 10.1038/s41684-024-01476-2 39548348 PMC11599037

[B28] Lazic S. E. Edmunds N. Pollard C. E. (2018). Predicting drug safety and communicating risk: benefits of a Bayesian approach. Toxicol. Sci. 162, 89–98. 10.1093/toxsci/kfx236 29126124

[B29] Li D. Garren J. Mangipudy R. Martin M. Tomlinson L. Vansell N. R. (2024). Statistical applications of virtual control groups to nonrodent animal toxicity studies: an initial evaluation. Regul. Toxicol. Pharmacol. 154, 105733. 10.1016/j.yrtph.2024.105733 39486783

[B30] Lin J. Gamalo-Siebers M. Tiwari R. (2019). Propensity-score-based priors for Bayesian augmented control design. Pharm. Stat. 18, 223–238. 10.1002/pst.1918 30537087

[B31] Ludbrook J. (2008). Outlying observations and missing values: how should they be handled? Clin. Exp. Pharmacol. Physiol. 35, 670–678. 10.1111/j.1440-1681.2007.04860.x 18215187

[B32] Noguchi K. Abel R. S. Marmolejo-Ramos F. Konietschke F. (2020). Nonparametric multiple comparisons. Behav. Res. Methods 52, 489–502. 10.3758/s13428-019-01247-9 31062191

[B33] OECD (2014). “Guidance document 116 on the conduct and design of chronic toxicity and carcinogenicity studies, supporting test guidelines 451, 452 and 453” in OECD series on testing and assessment, No. 116. Second edition. 10.1787/9789264221475-en

[B34] OJEU (2010). “Directive 2010/63/EU of the European Parliament and of the Council on the protection of animals used for scientific purposes,”, 53. OJEU, 33–79. Available online at: http://data.europa.eu/eli/dir/2010/63/oj (Accessed August 14, 2025).

[B35] Okamura T. Suzuki S. Ogawa T. Kobayashi J. Kusuoka O. Hatayama K. (2011). Background data for general toxicology parameters in RccHan™:WIST rats at 8, 10, 19 and 32 weeks of age. J. Toxicol. Pathol. 24, 195–205. 10.1293/tox.24.195 22319231 PMC3266354

[B36] Palazzi X. Anger T. L. Boulineau T. Grevot A. Guffroy M. Henson K. (2024). Points to consider regarding the use and implementation of virtual controls in nonclinical general toxicology studies. Regul. Toxicol. Pharmacol. 150, 105632. 10.1016/j.yrtph.2024.105632 38679316

[B37] Phuse (2024). Harmonization of SEND implementation to enable historical control data analysis. Available online at: https://advance.hub.phuse.global/wiki/spaces/WEL/pages/26806721/ (Accessed August 14, 2025).

[B38] Pinches M. D. Thomas R. Porter R. Camidge L. Briggs K. (2019). Curation and analysis of clinical pathology parameters and histopathologic findings from eTOXsys, a large database project (eTOX) for toxicologic studies. Regul. Toxicol. Pharmacol. 107, 104396. 10.1016/j.yrtph.2019.05.021 31128168

[B39] Ramaiah L. Arndt T. Thomas G. Shirai N. Sebastian M. Sims C. (2025). Toxicologic pathology forum*: opinion on the interpretation of statistical significance testing results from anatomic and clinical pathology data in nonclinical safety studies. Toxicol. Pathol. 53, 571–581. 10.1177/01926233251339113 40448609

[B40] Russell W. M. S. Burch R. L. (1959). The principles of humane experimental technique.

[B41] Sanz F. Pognan F. Steger-Hartmann T. Díaz C. Asakura S. Amberg A. (2023). eTRANSAFE: data science to empower translational safety assessment. Nat. Rev. Drug Discov. 22, 605–606. 10.1038/d41573-023-00099-5 37316648

[B42] Sato G. Nakajima M. Sakai K. Togashi Y. Yamamoto M. Inoue Y. (2024). Potential issues associated with the introduction of virtual control groups into non-clinical toxicology studies. Transl. Regul. Sci. 6, 1–9. 10.33611/trs.2023-009

[B43] Sellers R. S. Antman M. Phillips J. Khan K. N. Furst S. M. (2005). Effects of miglyol 812 on rats after 4 weeks of gavage as compared with methylcellulose/tween 80. Drug Chem. Toxicol. 28, 423–432. 10.1080/01480540500262839 16298873

[B44] Shannon C. E. (1948). A mathematical theory of communication. Bell Syst. Tech. J. 27, 379–423. 10.1002/j.1538-7305.1948.tb01338.x

[B45] Steger-Hartmann T. Kreuchwig A. Vaas L. Wichard J. Bringezu F. Amberg A. (2020). Introducing the concept of virtual control groups into preclinical toxicology testing. ALTEX 37, 343–349. 10.14573/altex.2001311 32242633

[B46] Steger-Hartmann T. Sanz F. Bringezu F. Soininen I. (2025a). IHI VICT3R: developing and implementing virtual control groups to reduce animal use in toxicology research. Toxicol. Pathol. 53, 1926233241303906. 10.1177/01926233241303906 39665320

[B47] Steger-Hartmann T. Duchateau-Nguyen G. Bringezu F. Onidi M. Stirn M. (2025b). Virtual control groups in non-clinical toxicology - a replicability challenge. ALTEX 42, 538–542. 10.14573/altex.2503061 40302301

[B48] Streiner D. L. (2018). Statistics commentary series: commentary No. 26: dealing with outliers. J. Clin. Psychopharmacol. 38, 170–171. 10.1097/JCP.0000000000000865 29578889

[B49] Vázquez-Borsetti P. (2024). Variability in rat weight gain during development. Lab. Anim. 58, 579–590. 10.1177/00236772241246370 39157979

[B50] Walley R. Sherington J. Rastrick J. Detrait E. Hanon E. Watt G. (2016). Using Bayesian analysis in repeated preclinical *in vivo* studies for a more effective use of animals. Pharm. Stat. 15, 277–285. 10.1002/pst.1748 27028721

[B51] Wang X. Suttner L. Jemielita T. Li X. (2022). Propensity score-integrated Bayesian prior approaches for augmented control designs: a simulation study. J. Biopharm. Stat. 32, 170–190. 10.1080/10543406.2021.2011743 34939894

[B52] Wright P. S. R. Smith G. F. Briggs K. A. Thomas R. Maglennon G. Mikulskis P. (2023). Retrospective analysis of the potential use of virtual control groups in preclinical toxicity assessment using the eTOX database. Regul. Toxicol. Pharmacol. 138, 105309. 10.1016/j.yrtph.2022.105309 36481280

[B53] Yu R. (2023). “Matching methods for large observational studies,” in Handbook of matching and weighting adjustments for causal inference (New York, United States of America: CRC Press), 239–260. 10.1201/9781003102670-13

