# Supplementary material for: Analysis of rat toxicology studies: statistical agreement between virtual and concurrent controls in detecting treatment effects on liver enzymes
Source: Front Pharmacol. 2026 Apr 30;17:1704002. doi: 10.3389/fphar.2026.1704002 (PMC13172619; doi:10.3389/fphar.2026.1704002)
Supplement: Supplementary file 1 [file Supplementaryfile1.docx]

### SUPPLEMENTARY MATERIALS

**Supplementary Table 1:** The lower and upper limits of the reference ranges for ALT, AST and ALP in each sex are displayed in the three last columns, numbered 1 to 3. They are computed with the following approaches: column 1) arbitrary bounds at the 2.5th and 97.5th percentiles; column 2) mean ± three times the SD (between-study variability) extracted from a mixed-effects model (maximum likelihood estimates) of the enzyme activity level as a function of “sex”, with study as a random effect; column 3) the 2.5th and 97.5th percentiles of the fitted distribution obtained from a Box-Cox transformation model (Ammer et al., 2021). For each enzyme and each sex the lower and upper limits are displayed on two rows. ALP, alkaline phosphatase; ALT, alanine aminotransferase; AST, aspartate aminotransferase; F, female; M, male; SD, standard deviation.

| **Test** | **Sex** | **Lower/upper**  **limit** | **1** | **2** | **3** |
| --- | --- | --- | --- | --- | --- |
| Alanine Aminotransferase_S | F | Lower | 0.250 | 0.026 | 0.250 |
| Alanine Aminotransferase_S | F | Upper | 1.034 | 1.109 | 1.042 |
| Alanine Aminotransferase_S | M | Lower | 0.333 | 0.171 | 0.326 |
| Alanine Aminotransferase_S | M | Upper | 1.286 | 1.254 | 1.154 |
| Alkaline Phosphatase_S | F | Lower | 0.574 | -0.327 | 0.607 |
| Alkaline Phosphatase_S | F | Upper | 3.144 | 3.267 | 2.690 |
| Alkaline Phosphatase_S | M | Lower | 1.250 | 0.904 | 1.284 |
| Alkaline Phosphatase_S | M | Upper | 5.060 | 4.498 | 4.714 |
| Aspartate Aminotransferase_S | F | Lower | 1.010 | 0.199 | 1.038 |
| Aspartate Aminotransferase_S | F | Upper | 2.531 | 2.945 | 2.331 |
| Aspartate Aminotransferase_S | M | Lower | 1.050 | 0.262 | 1.069 |
| Aspartate Aminotransferase_S | M | Upper | 2.498 | 3.008 | 2.469 |

### Supplementary Table 2. Outliers identified in CCGs in the 40 reanalyzed studies, for each enzyme, in females (F) and males (M). The distribution of each enzyme activity level in the CCG (blue) and VCG (yellow) are displayed separately for each sex. The reference intervals are shown in between parentheses for each enzyme and sex (in μkat/L). ALP, alkaline phosphatase; ALT, alanine aminotransferase; AST, aspartate aminotransferase; CCG, concurrent control group; VCG, virtual control groups.

| **Study** | **Presence of outliers in CCG** | | | **Distribution of data** | | | | | |
| --- | --- | --- | --- | --- | --- | --- | --- | --- | --- |
| ID | ALT (M/F) | ALP (M/F) | AST (M/F) | ALT (M) (0.33–1.15) | ALT (F)  (0.25–1.04) | ALP (M) (1.28–4.71) | ALP (F) (0.61–2.69) | AST (M) (1.07–2.47) | AST (F) (1.04–2.33) |
| 8647 | -- / 1 | -- / -- | -- / -- | 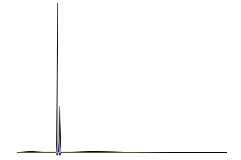 | 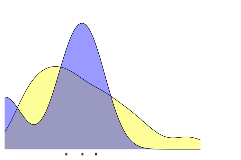 | 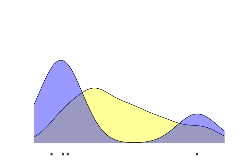 | 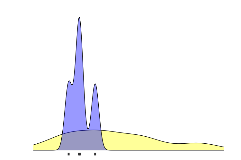 | 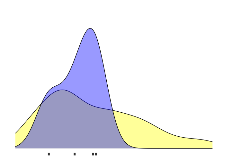 | 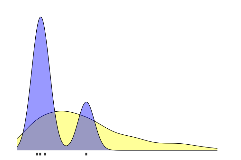 |
| 8737 | -- / -- | 1 / -- | -- / -- | 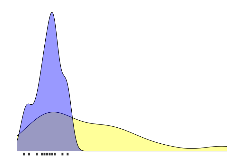 | 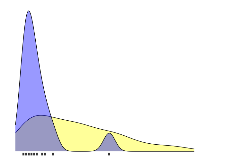 | 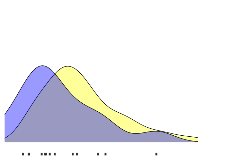 | 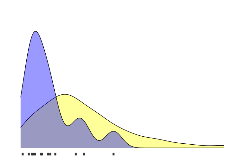 | 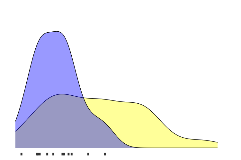 | 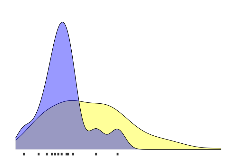 |
| 8893 | -- / -- | -- / -- | -- / -- | 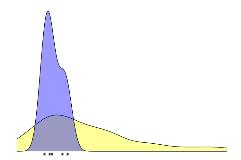 | 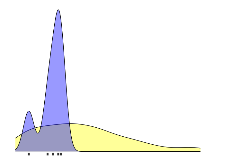 | 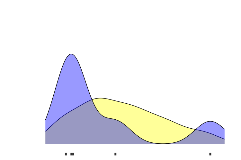 | 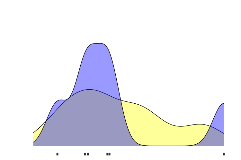 | 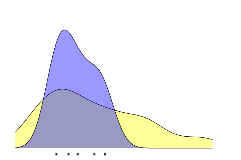 | 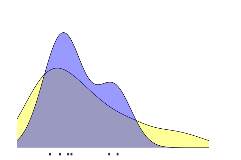 |
| 8895 | -- / 1 | -- / -- | -- / -- | 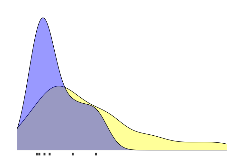 | 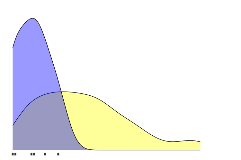 | 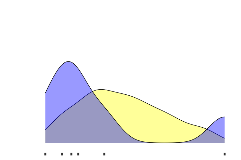 | 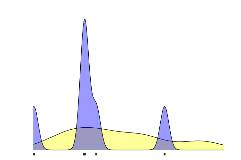 | 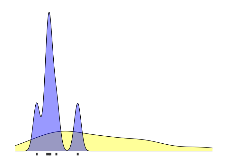 | 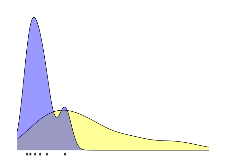 |
| 8975 | 1 / -- | -- / -- | -- / 1 | 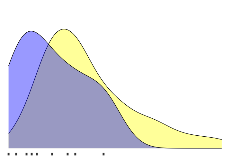 | 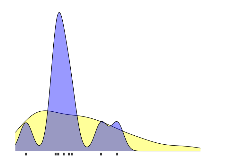 | 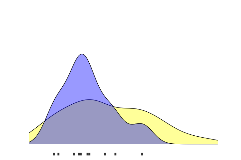 | 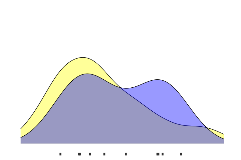 | 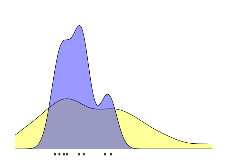 | 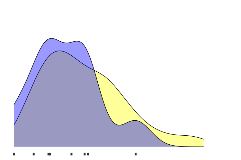 |
| 9114 | -- / -- | -- / -- | -- / -- | 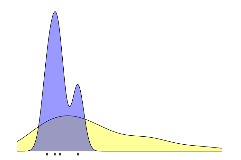 | 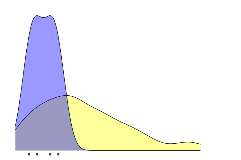 | 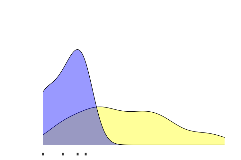 | 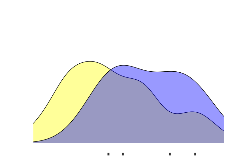 | 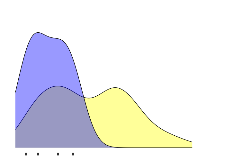 | 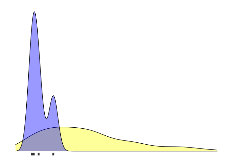 |
| 9477 | -- / 1 | -- / -- | -- / 3 | 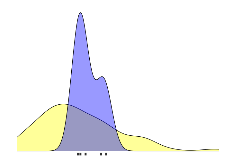 | 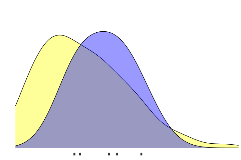 | 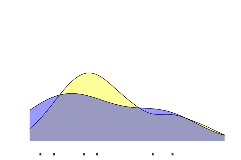 | 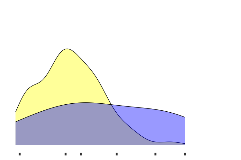 | 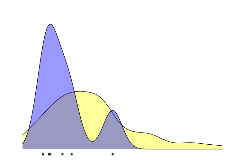 | 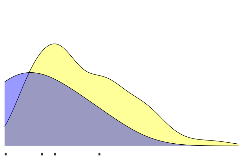 |
| 9557 | -- / -- | -- / -- | -- / -- | 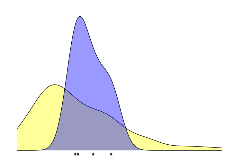 | 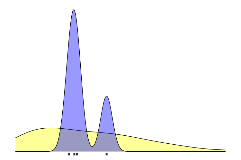 | 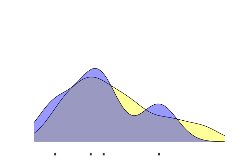 | 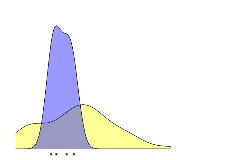 | 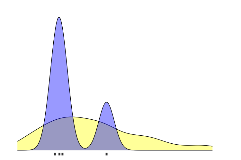 | 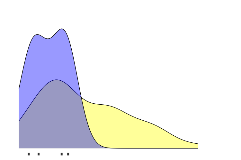 |
| 9576 | -- / -- | 1 / -- | 1 / 1 | 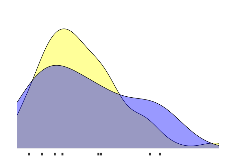 | 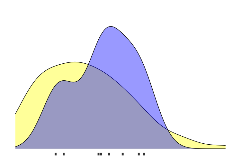 | 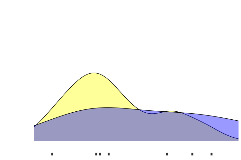 | 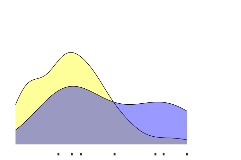 | 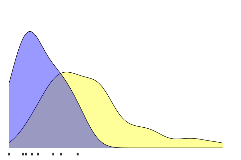 | 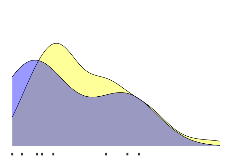 |
| 10574 | 1 / -- | -- / -- | 1 / -- | 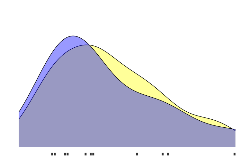 | 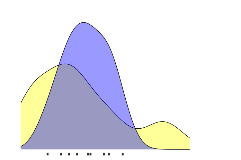 | 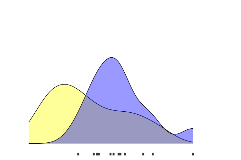 | 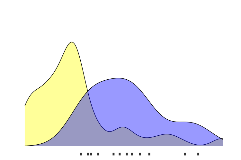 | 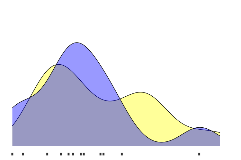 | 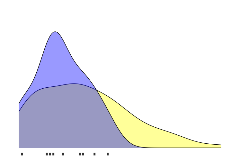 |
| 10594 | -- / -- | -- / 5 | -- / -- | 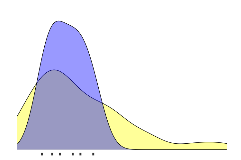 | 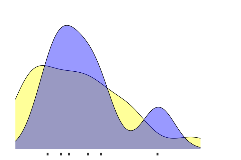 | 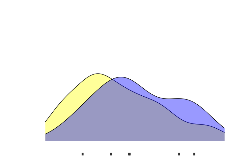 | 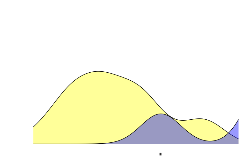 | 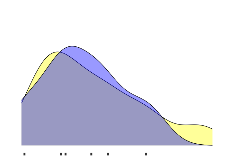 | 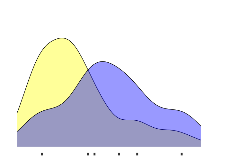 |
| 10696 | -- / -- | -- / -- | -- / -- | 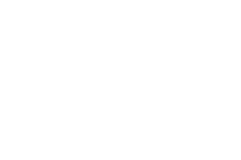 | 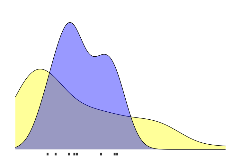 | 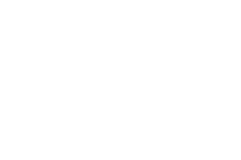 | 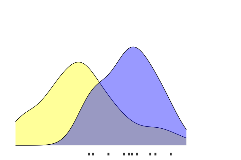 | 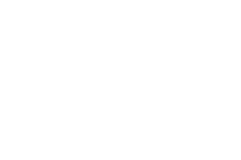 | 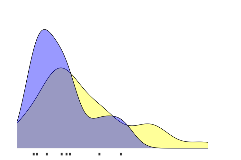 |
| 10704 | -- / -- | -- / -- | -- / -- | 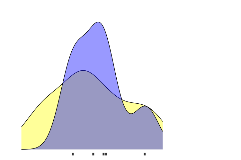 | 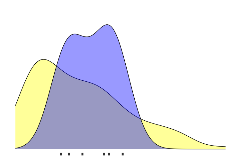 | 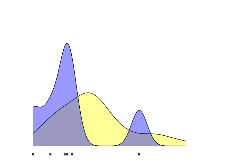 | 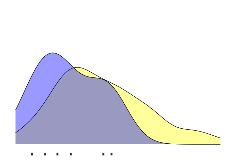 | 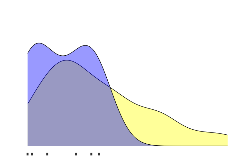 | 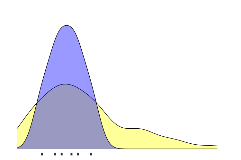 |
| 13859 | -- / -- | -- / -- | 3 / 5 | 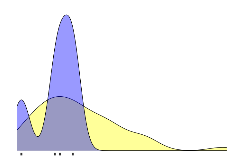 | 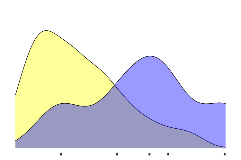 | 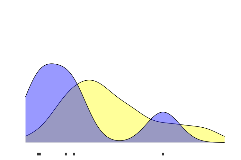 | 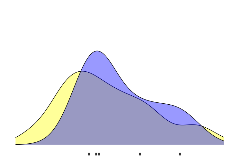 | 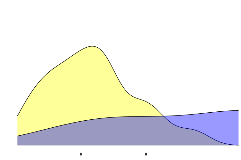 | 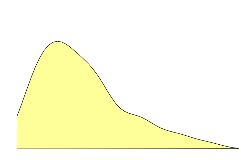 |
| 13893 | -- / -- | -- / -- | 4 / 4 | 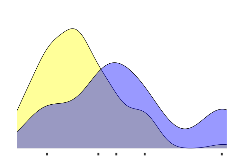 | 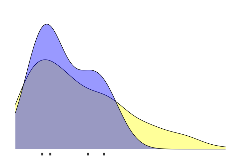 | 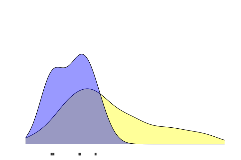 | 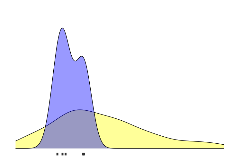 | 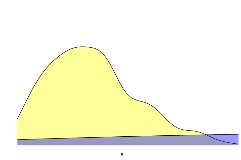 | 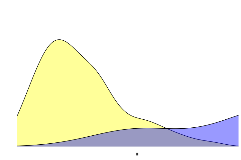 |
| 17192 | -- / -- | -- / -- | 1 / -- | 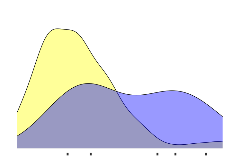 | 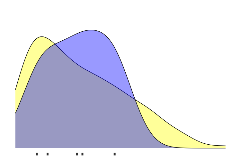 | 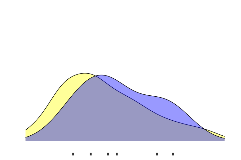 | 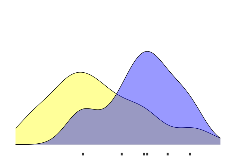 | 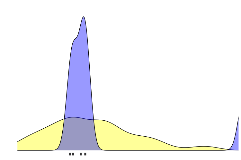 | 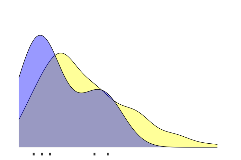 |
| 17193 | -- / -- | -- / -- | -- / -- | 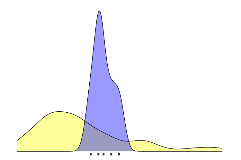 | 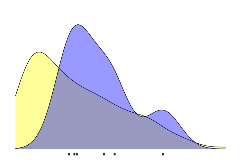 | 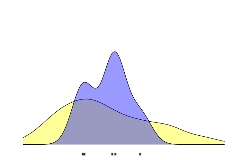 | 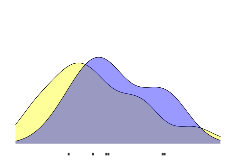 |  |  |
| 17261 | 1 / -- | -- / 1 | 2 / -- |  |  |  |  |  |  |
| 17271 | -- / -- | -- / -- | -- / -- |  |  |  |  |  |  |
| 17289 | -- / 1 | -- / -- | -- / -- |  |  |  |  |  |  |
| 17341 | -- / 1 | -- / -- | -- / 3 |  |  |  |  |  |  |
| 17372 | -- / -- | -- / -- | -- / 1 |  |  |  |  |  |  |
| 17424 | -- / -- | -- / -- | -- / 2 |  |  |  |  |  |  |
| 18661 | 1 / -- | -- / -- | -- / -- |  |  |  |  |  |  |
| 19002 | -- / -- | -- / -- | -- / -- |  |  |  |  |  |  |
| 20165 | -- / -- | -- / 1 | -- / -- |  |  |  |  |  |  |
| 21740 | 2 / -- | -- / -- | 1 / -- |  |  |  |  |  |  |
| 21742 | -- / -- | -- / -- | -- / 1 |  |  |  |  |  |  |
| 21784 | -- / -- | -- / -- | 1 / -- |  |  |  |  |  |  |
| 21830 | -- / -- | -- / -- | -- / -- |  |  |  |  |  |  |
| 21839 | -- / -- | -- / -- | 2 / 2 |  |  |  |  |  |  |
| 21841 | -- / -- | -- / -- | 2 / -- |  |  |  |  |  |  |
| 21843 | -- / -- | -- / -- | -- / 1 |  |  |  |  |  |  |
| 21855 | -- / -- | 1 / -- | 1 / 1 |  |  |  |  |  |  |
| 22730 | 1 / 2 | 1 / -- | -- / -- |  |  |  |  |  |  |
| 22802 | -- / -- | 3 / -- | -- / 3 |  |  |  |  |  |  |
| 60025 | -- / 2 | -- / -- | -- / -- |  |  |  |  |  |  |
| 157449 | 1 / 1 | -- / -- | -- / 1 |  |  |  |  |  |  |
| 592676 | -- / -- | -- / -- | -- / 1 |  |  |  |  |  |  |
| 827977 | -- / -- | -- / -- | -- / -- |  |  |  |  |  |  |

**Supplementary Table 3.** The normality of CCG data in the 40 reanalyzed studies, for each enzyme, in females (F) and males (M), and the percentage of samples with normally distributed data among the 100 VCGs. ALP, alkaline phosphatase; ALT, alanine aminotransferase; AST, aspartate aminotransferase; CCG, concurrent control group; N, no; NA, not available; VCG, virtual control groups; Y, yes.

| **Study ID** | **ALT CCG/VCG Female** | **ALT CCG/VCG  Male** | **ALP CCG/VCG Female** | **ALP CCG/VCG  Male** | **AST CCG/VCG Female** | **AST CCG/VCG  Male** |
| --- | --- | --- | --- | --- | --- | --- |
| 8647 | Y / 93 % | N / 90 % | Y / 89 % | N / 96 % | N / 94 % | Y / 95 % |
| 8737 | N / 77 % | Y / 76 % | N / 67 % | Y / 81 % | Y / 93 % | Y / 91 % |
| 8893 | Y / 95 % | Y / 89 % | Y / 92 % | N / 94 % | Y / 93 % | Y / 97 % |
| 8895 | Y / 96 % | Y / 86 % | Y / 96 % | N / 99 % | Y / 89 % | Y / 91 % |
| 8975 | Y / 89 % | Y / 78 % | Y / 84 % | Y / 93 % | Y / 93 % | Y / 96 % |
| 9114 | Y / 94 % | Y / 94 % | Y / 94 % | Y / 99 % | Y / 97 % | Y / 98 % |
| 9477 | N / 97 % | Y / 89 % | Y / 93 % | Y / 91 % | N / 92 % | Y / 91 % |
| 9557 | Y / 97 % | Y / 93 % | Y / 97 % | Y / 92 % | Y / 95 % | N / 90 % |
| 9576 | Y / 98 % | Y / 90 % | Y / 98 % | Y / 91 % | Y / 87 % | Y / 85 % |
| 10574 | Y / 87 % | N / 99 % | Y / 45 % | Y / 86 % | Y / 90 % | Y / 73 % |
| 10594 | Y / 95 % | Y / 89 % | Y / 97 % | Y / 96 % | Y / 90 % | Y / 93 % |
| 10696 | Y / 76 % | NA / NA | Y / 97 % | NA / NA | Y / 77 % | NA / NA |
| 10704 | Y / 91 % | Y / 98 % | Y / 92 % | Y / 92 % | Y / 89 % | Y / 93 % |
| 13859 | Y / 94 % | Y / 95 % | Y / 97 % | Y / 93 % | Y / 88 % | Y / 97 % |
| 13893 | Y / 92 % | Y / 91 % | Y / 94 % | Y / 89 % | Y / 95 % | Y / 96 % |
| 17192 | Y / 90 % | Y / 91 % | Y / 91 % | Y / 97 % | Y / 93 % | N / 97 % |
| 17193 | Y / 94 % | Y / 95 % | Y / 93 % | Y / 96 % | N / 90 % | Y / 95 % |
| 17261 | Y / 87 % | Y / 80 % | Y / 97 % | Y / 87 % | Y / 85 % | Y / 77 % |
| 17271 | N / 86 % | Y / 92 % | N / 85 % | Y / 98 % | Y / 89 % | N / 89 % |
| 17289 | N / 95 % | NA / NA | Y / 91 % | NA / NA | Y / 96 % | NA / NA |
| 17341 | N / 63 % | Y / 90 % | Y / 88 % | Y / 93 % | Y / 85 % | Y / 84 % |
| 17372 | Y / 93 % | N / 95 % | Y / 92 % | Y / 97 % | N / 98 % | Y / 92 % |
| 17424 | Y / 92 % | Y / 94 % | Y / 94 % | Y / 96 % | Y / 93 % | Y / 95 % |
| 18661 | Y / 97 % | Y / 93 % | Y / 97 % | Y / 98 % | N / 94 % | Y / 94 % |
| 19002 | Y / 98 % | Y / 95 % | Y / 95 % | Y / 95 % | Y / 91 % | Y / 92 % |
| 20165 | Y / 89 % | N / 90 % | N / 87 % | Y / 89 % | Y / 86 % | N / 94 % |
| 21740 | Y / 94 % | Y / 95 % | Y / 96 % | Y / 98 % | Y / 94 % | Y / 94 % |
| 21742 | Y / 90 % | NA / NA | Y / 85 % | NA / NA | Y / 92 % | NA / NA |
| 21784 | Y / 95 % | Y / 92 % | Y / 96 % | Y / 95 % | Y / 96 % | Y / 89 % |
| 21830 | NA / NA | Y / 94 % | NA / NA | Y / 98 % | NA / NA | Y / 96 % |
| 21839 | Y / 87 % | Y / 73 % | Y / 88 % | Y / 93 % | Y / 88 % | Y / 98 % |
| 21841 | Y / 96 % | Y / 94 % | Y / 92 % | N / 95 % | Y / 90 % | Y / 94 % |
| 21843 | N / 93 % | NA / NA | Y / 93 % | NA / NA | N / 91 % | NA / NA |
| 21855 | Y / 91 % | Y / 92 % | N / 95 % | Y / 73 % | Y / 90 % | Y / Y |
| 22730 | Y / 77 % | Y / 74 % | Y / 69 % | Y / 87 % | Y / 92 % | Y / 84 % |
| 22802 | Y / 88 % | Y / 81 % | Y / 94 % | Y / 83 % | Y / 94 % | Y / 97 % |
| 60025 | Y / 94 % | Y / 93 % | Y / 95 % | Y / 93 % | Y / 91 % | N / 95 % |
| 157449 | N / 89 % | Y / 94 % | Y / 93 % | Y / 96 % | N / 92 % | Y / 94 % |
| 592676 | Y / 96 % | NA / NA | Y / 88 % | NA / NA | Y / 92 % | NA / NA |
| 827977 | Y / 97 % | NA / NA | Y / 94 % | NA / NA | Y / 94 % | NA / NA |

**Supplementary Table 4.** Cases of full disagreement (i.e. none of the effect sizes computed with the 100 sampled VCGs fell into the confidence interval of the effect size computed with CCG). Full disagreement is observed in 10 studies in one or several enzymes, in different dose levels. The * indicates there is also full disagreement observed with h-VCG. ALP, alkaline phosphatase; ALT, alanine aminotransferase; AST, aspartate aminotransferase; Hi, high; Lo, low; Mi, medium.

|  | **Female** | | | **Male** | | |  |
| --- | --- | --- | --- | --- | --- | --- | --- |
| **Study** | **ALT** | **ALP** | **AST** | **ALT** | **ALP** | **AST** | **Total** |
| 8737 |  |  |  | Hi |  |  | 1 |
| 9114 |  |  |  |  | Hi |  | 1 |
| 10594 |  | Mi, Hi |  |  |  |  | 2 |
| 13859 |  |  | Lo, Mi, Hi* |  |  |  | 3 |
| 13893 |  |  |  |  |  | Lo, Mi | 2 |
| 17261 |  |  |  | Lo |  |  | 1 |
| 18661 |  |  |  |  |  | Mi | 1 |
| 21740 |  |  |  | Lo |  |  | 1 |
| 22802 |  |  | Hi |  | Lo, Mi, Hi |  | 4 |
| 157449 | Hi |  |  |  |  |  | 1 |
| **Total** | **1** | **2** | **4** | **3** | **4** | **3** | **17** |

**Supplementary Figure 1**

**A)**

**B)**

**C)**

**D)**

**E)**

Power for detecting differences in ALT, AST and ALP activity levels between the treated and control groups, as a function of the ratio of VCG to CCG sample sizes (at a significance level of 0.05) in females and males per dose group in each reanalyzed study. Triangles indicate the power of the comparison using the CCG as control. A line depicts the power of the comparison using VCGs as control. ALP, alkaline phosphatase; ALT, alanine aminotransferase; AST, aspartate aminotransferase; CCG, concurrent control group; Hi, high; Lo, low; Mi, medium; VCG, virtual control groups.
